# Delineation of the reservoir petrophysical parameters from well logs validated by the core samples case study Sitra field, Western Desert, Egypt

**DOI:** 10.1038/s41598-024-77371-0

**Published:** 2024-11-05

**Authors:** Hadeel Mohamed, Walid M. Mabrouk, Ahmed Metwally

**Affiliations:** Geophysics Department, Faculty of Science, Cairo University, Giza, 12613 Egypt

**Keywords:** Abu EL-Gharadig Basin, Reservoir evaluation, Abo Roash F & G, North Western Desert, Unconventional reservoir, Geology, Geophysics

## Abstract

In the northern section of the Western Desert, there are many extremely profitable petroleum and natural gas deposits in the Abu EL-Gharadig Basin. This study aims to highlight the hydrocarbon potential of Abu Roash F Formation, which stands for high organic content unconventional tight reservoirs, and Abu Roash G Formation which stands for conventional sand reservoirs, in Sitra field located in the central-western part of the Abu EL-Gharadig Basin. The research employed well-log data from four wells to ascertain petrophysical properties combined with core samples of two wells for a comprehensive examination and description of lithology. Initially, we commenced the execution of petrophysical analysis, encompassing log quality control procedures. Subsequently, we identified and revealed zones of interest and hydrocarbon indicators in both formations. Additionally, we ascertained the three most influential parameters, shale Volume, effective Porosity, and water saturation, which serve as defining factors for reservoir quality. Subsequently, an examination of the core samples, which encompassed lithologic description, lithofacies analysis, paleoenvironmental interpretation, petrographic analysis, and porosity assessment is conducted. For the sake of a more accurate interpretation, we conclude our research with cartographic maps created to evaluate the geographical distribution of hydrocarbon potential based on petrophysical characteristics, Distribution of the net-to-gross ratio among wells by correlating the litho-saturation models (rock models) for the four wells. The foregoing results declare that The Abu Roash F carbonate-rich rocks are a contender for unconventional tight oil reservoir potential with thin secondary porosity and high organic content, which normally requires a kind of hydraulic fracturing for prospective oil extraction, Furthermore, the upper section of Abu Roash G formation, particularly in well sitra8-03, has highly favorable conventional reservoir characteristics.

## Introduction

Significant oil and gas discoveries have recently occurred in Egypt’s Western Desert, and these discoveries have been reported to be very promising. In the northern part of the Western Desert, there are several extremely productive oil and gas resources in the Abu EL-Gharadig Basin^1–3^. The basin is characterized by its sedimentary fill, consisting of various rock layers deposited over millions of years^4–8^. This research aims to evaluate wireline logging data of Abu Roash G & Abu Roash F formations in the Abu EL-Gharadig basin Western Desert to detect probably bypassed hydrocarbon intervals. As Abu Roash G Member is extensively dispersed in the northern Western Desert Basins, exhibiting significant thickness (220–1000 m) and significant hydrocarbon concentration^9^, and Abu Roash F is the most advantageous source rock in the northern Western Desert basins, exhibiting organic richness with total organic carbon content (TOC) between 1.5% and 6%^10–14^. The western desert region has undergone a tectonic history marked by both compressional and extensional forces, Secondary porosity may arise from extensional forces within the Abu Roash F formation, positioning it as a potential unconventional tight oil reservoir that may require hydraulic fracturing or nitrogen injection in an underbalanced system for commercial oil production, similar to the Buda Limestone formation in Texas^10,11,15^.

### The study area

**Fig. 1:**
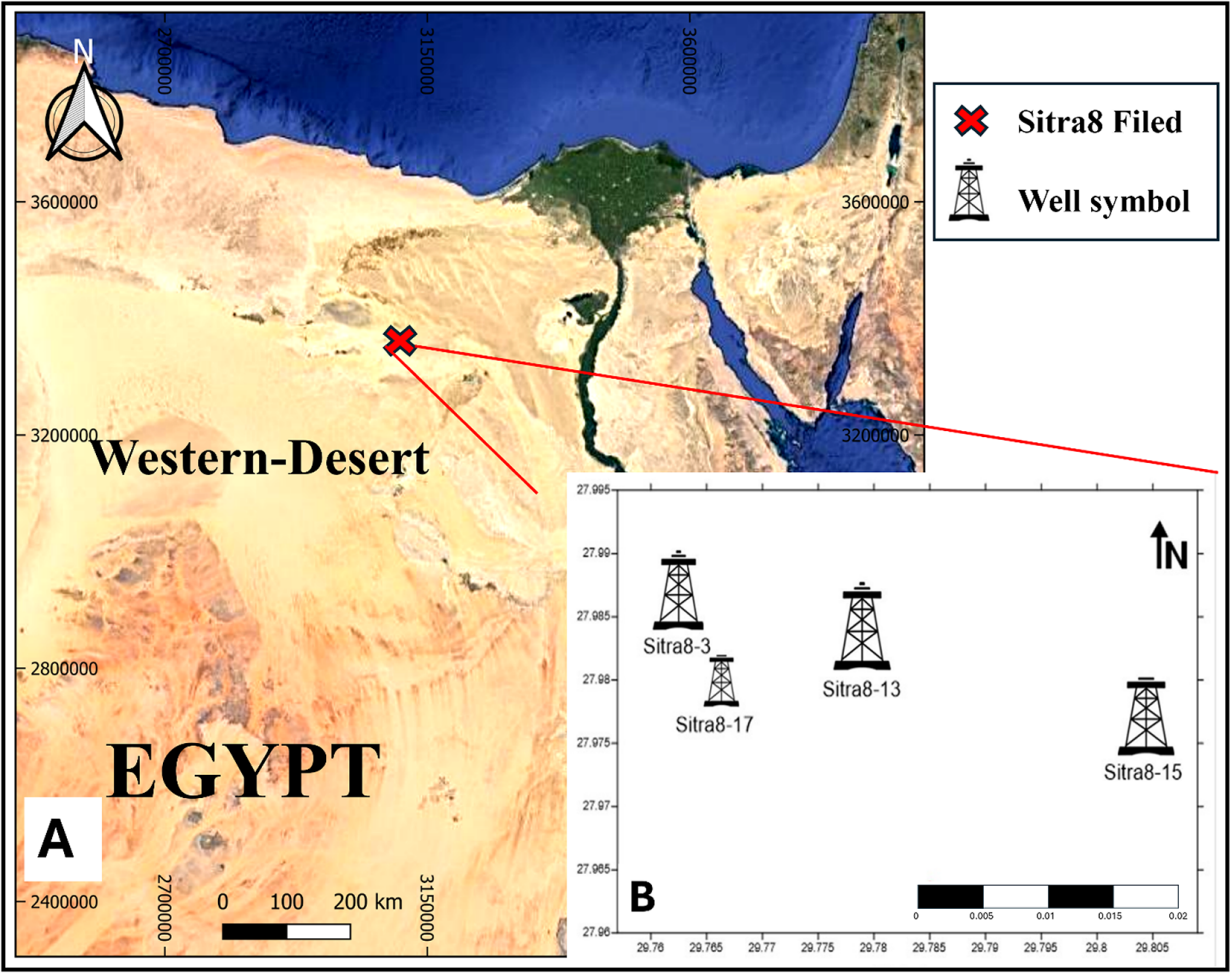
(**A**) Location map of study area created by QGIS software, (**B**) Location Map created by surfer 13 software of the four studied wells in Sitra field.

This paper focuses on the study of the Sitra field. Badr El Din Petroleum Company manages Sitra concession—Bapetco operates on behalf of the Sitra Petroleum Company (SiPetCo)^3^. Figure 1 shows the location of the Sitra concession, which extends over 322.4 km^2^ in the western desert of Egypt’s northern region, specifically in the central-western part of the Abu EL-Gharadig Basin. The Sitra Field region is comprised of several structural closures. More than 39 wells have been drilled in the Sitra 8 block^3,16^, which serves as the primary producing closure, since 1993.The most significant section in the Sitra Field that produces hydrocarbons is the Abu Roash “G” Member^16–18^.

## Geological setting and structure controlling the area

Located in Egypt’s North Western Desert, the Abu EL-Gharadig Basin is approximately 330 km long and 50–75 km broad, making it a major Mesozoic extensional rift basin^17^. The Abu Gharadiq Basin is a prototypical half-graben characterized by transverse normal faults that interrupt a network of linear horsts and depressions^19^. The main faults in the Abu EL-Gharadig basin run along two main lines: NW-SE and WSW-ENE. These lines describe the structural trends of the Tethyan and Syrian Arcs, respectively. The formation of these faults was caused by stretching the crust during the Early Cimmerian rifting process. This was caused by oblique dextral strike-slip movement between the Eurasian and African plates, along with apparent sinistral displacement along transform faults that lined up the horst and grabens within the basin^19,20^. During the Early Cretaceous-Santonian period, the African Plate moved towards the north, causing significant changes in the subsurface sedimentary layers of the northern Western Desert^4–8^. This movement resulted in extensive inversion tectonics. The inversion phase had an impact on the previous normal faults in the Abu EL-Gharadig Basin. As a result of this phase, a series of folds formed in an NE-SW anticline direction, known as the Syrian arc fold system^21^.

## Stratigraphy framework

**Fig. 2:**
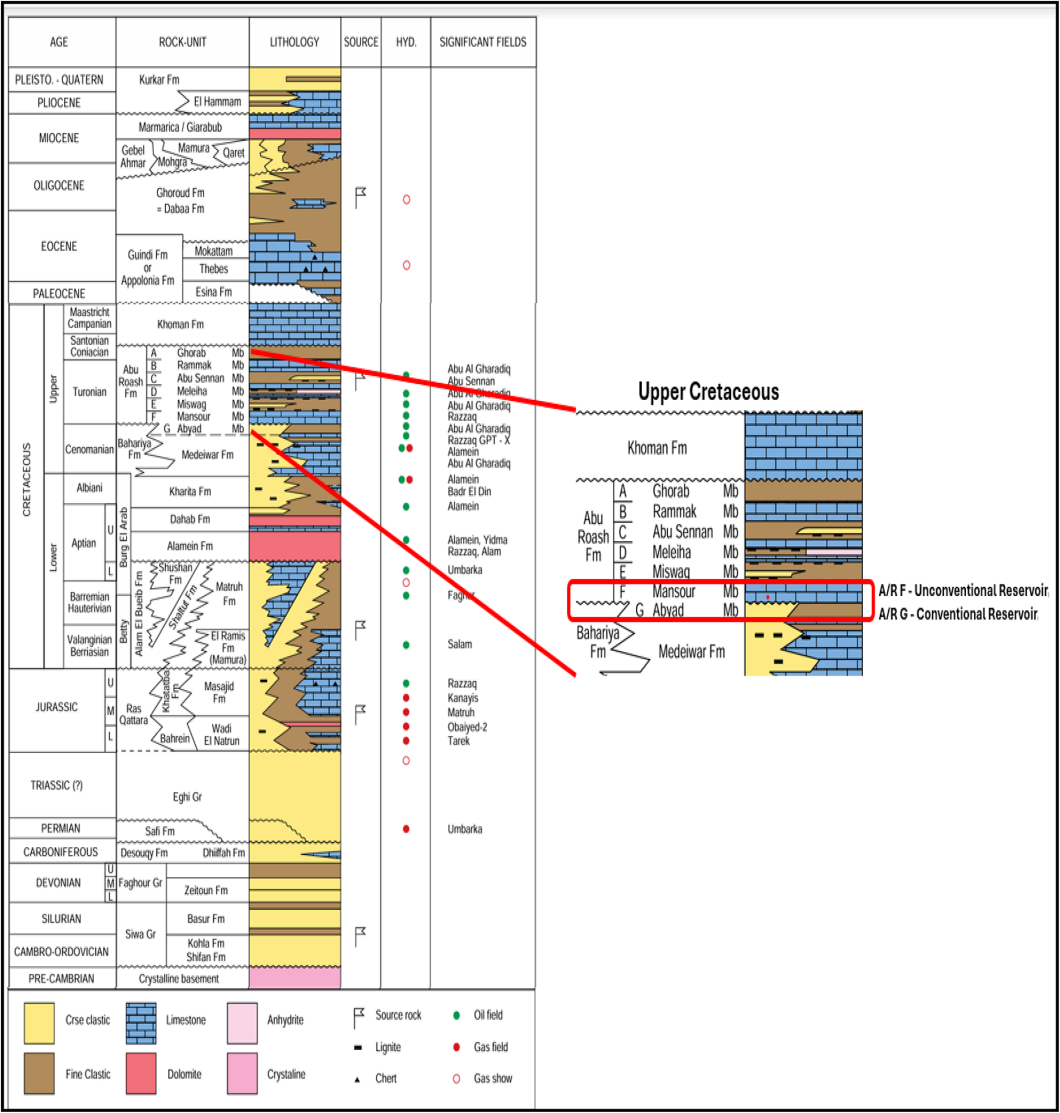
Stratigraphic column of Western Desert^4,22^, showing upper cretaceous sequence emphasizing Abu Roash F and G.

The present research is predominately concerned with Late Cretaceous formations which are when the Abu EL-Gharadig Basin originated. In the Abu El-Gharadig Basin, the sedimentary cover, denoting the layers of deposits atop the basement rocks, increases in thickness towards the north, attaining over 35,000 ft before gradually diminishing to 9,800 ft over the Ras Qattara ridge, which constitutes the northern boundary of the basin^3,16,20,22^. According to litho-stratigraphic classification, the Upper Cretaceous rocks are categorized into three principal units: the Bahariya Formation, the Abu-Roash Formation, and the Khoman Formation, listed from lowest to highest (Fig. 2). The Abu-Roash Formation predominantly consists of clastic and carbonate rocks arranged in alternating strata indicative of regressive and transgressive phases, divided into seven separate groups from top to bottom, labeled “A,” “B,” “C,” “D,” “E,” “F,” and “G”^16,23^. The stratigraphic stages of the Cretaceous sand and sandstone reservoirs commence with the Albian Kharita sands, progress to the Early Cenomanian Bahariya sands, and culminate with the Turonian Abu Roash “C” and “G” sands as reservoir rocks^24^. High bituminous clay–carbonate rocks are present in most Abu Roash formations in the Abu EL-Gharadig Basin, particularly the Abu Roash (F) Member^4–8^.

## Data and methodology

To accomplish the primary objectives of the current paper, the wire-line log data (Gamma Ray, Density, Neutron, Sonic, and Resistivity) provided by Badr EL-Din Company were examined using interactive petrophysics software (Techlog.2015) to determine petrophysical characteristics for wells (Sitra8-03, Sitra8-13, Sitra8-15 & Sitra8-17), Integrated with core samples of Abu Roash G formation exist in (Sitra8-3, & Sitra8-15) wells for detailed lithologic description and analysis. The outlines of this case study are provided below.

1. Petrophysical analysis:
  Log quality control and log display.
  Zone of interest & lithology identification.
  Hydrocarbon indicators.
  Picket plot.
  Petrophysical parameters (Vsh, φeff, SW & bulk volume of water BVW).
2. Core analysis.
3. Interpretation (mapping & correlation).

### Petrophysical analysis

#### Log quality control (LQC)

Log quality control in petrophysics is an essential process to ensure the accuracy and reliability of well-log data. It involves various steps and techniques to identify and address any issues or spikes in the acquired log data^5,25–27^. This process involves (1) Log Header Review, which includes rescaling and checking for proper depth measurements, units, well identification, and other relevant metadata for each log. (2) Depth-matching techniques are employed to accurately align the data from different logs. (3) Calibration Log data against known standards or reference data to correct any systematic errors or biases^28^. (4) Data Editing and filtering for any outliers or data points that do not meet certain quality criteria. Data points may be edited or removed from the dataset to ensure data integrity. (5) Wash out and mud cake which is used as indicators for the permeable zone can be identified by caliber log and bit size. (6) Shale parameters determination (ρsh, ϕsh, Δtsh, GRsh, and Rtsh), which can be readily obtained from the thick shale zone^29^. The preceding steps were implemented on our data.

A specific correction has been implemented to A/R F formation to eliminate the influence of organic matter present. Organic matter exhibits reduced resistivity compared to the adjacent mineral matrix owing to its conductivity. This may result in erroneous resistivity measurements if not adequately rectified. The porosity log may also be affected. The complication stems from the total porosity readings from logs potentially encompassing both matrix porosity and porosity attributed to organic matter. We employed Shale Correction Models to quantify the influence of organic matter on resistivity to mitigate these effects. These models utilize empirical correction factors, and the effect of organic matter in the porosity log was corrected by subtracting the contributions of organic matter from the total porosity^11,30,31^.

### Log display

**Fig. 3:**
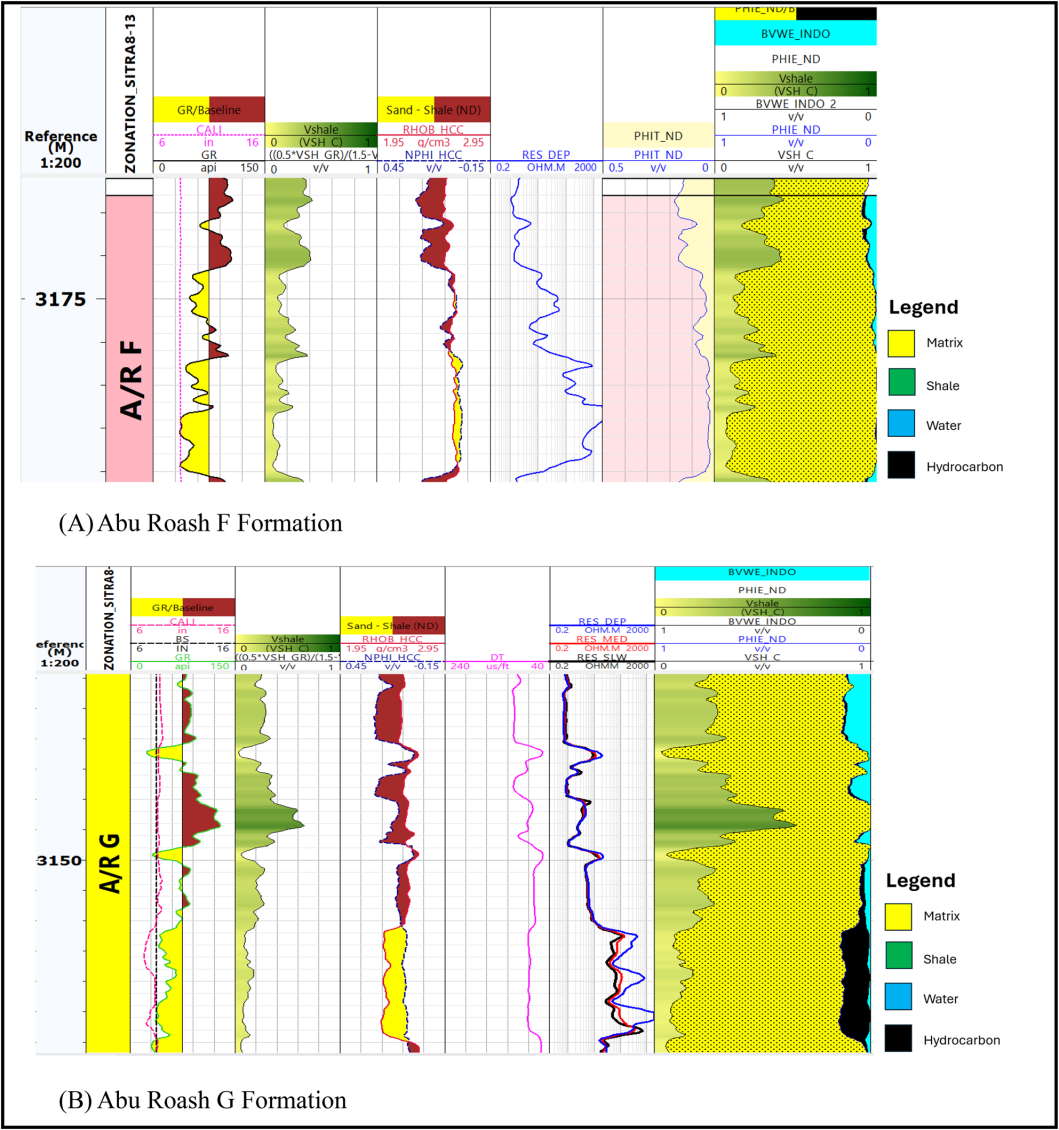
Log display shows log quality control application on (**A**) Abu Roash F formation and (**B**) Abu Roash G formation.

The well log data presented in (Fig. 3) is the result of implementing log quality control for the well (Sitra8-3). The LQC is applied for all the available well data.

### Zone of interest and lithology identification

**Fig. 4:**
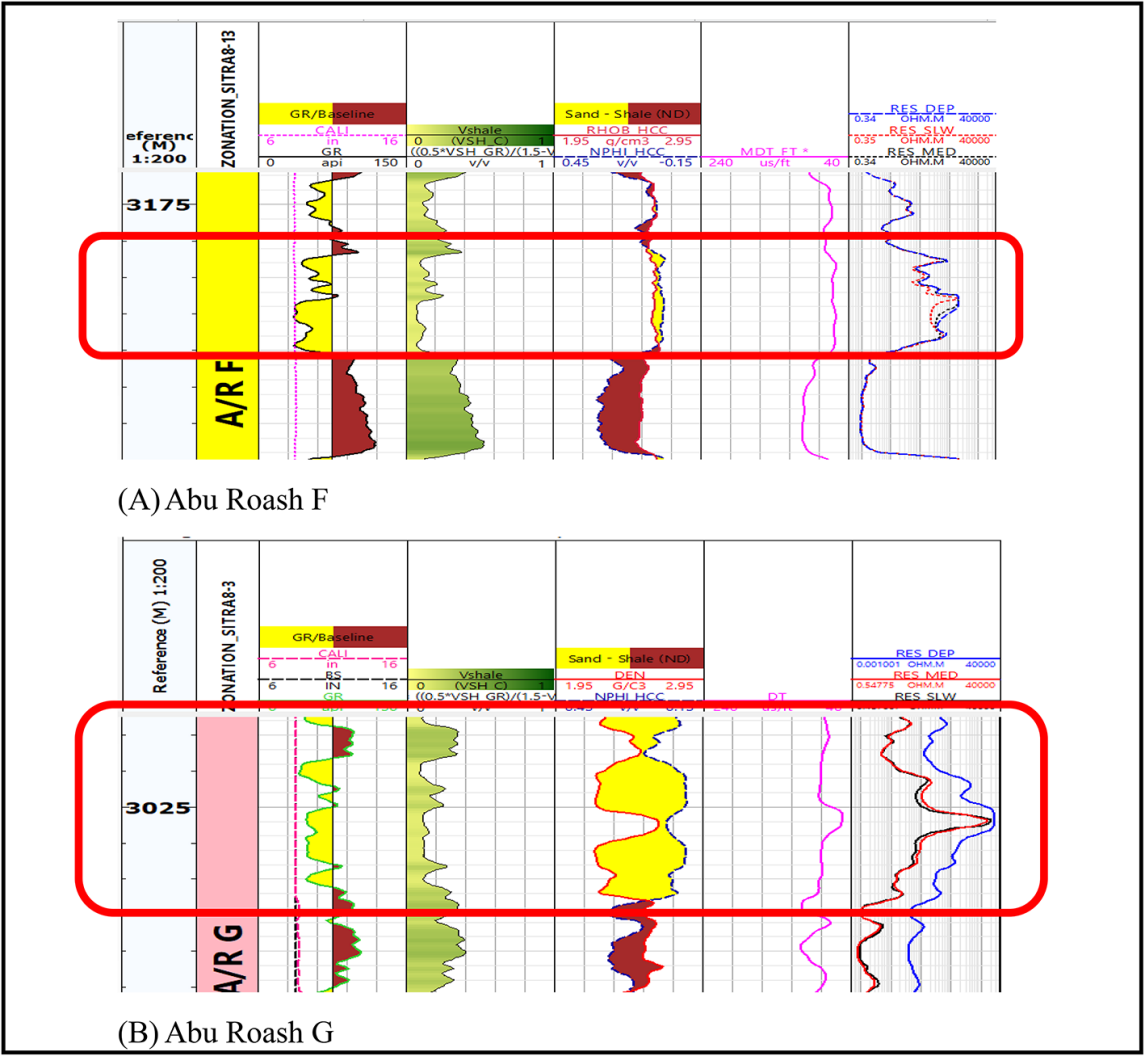
(**A**) Highlighted potential zone of interest in AR/F (un-conventional reservoir). (**B**) Highlighted zone of interest in AR/G (conventional reservoir).

Following the implementation of the log quality control procedure, we can now identify zones of interest that have been selected to achieve the overarching purpose of this research (Fig. 4). The primary determinant in conventional reservoirs (AR/G) is the augmentation of the deep resistivity log, which indicates the presence of hydrocarbon. Additionally, the zone should exhibit reduced gamma ray reactions and the crossovers of density-neutron logs, which indicate the presence of a porous zone, while the most crucial parameters in the unconventional reservoir (Carbonate A/R F) is an increase in resistivity, low gamma ray, and a coincident density and neutron response because of the tightness of the reservoir (Fig. 4). In the investigated wells, the lithological composition of the Abu Roash G & F formations was determined in Table 1 by combining Density-resistivity/Neutron-Resistivity overlays and various log cross-plots, including density–neutron cross-plots, sonic-neutron cross-plot, M-N plot, and MID plot.

**Table 1:** Displays the outcomes derived from the cross-plots.

Tool/formation	Abo Roash F	Abo Roash G
Density-resistivity/neutron-resistivity overlays	Carbonate	Clastic
Density–neutron cross plot	Limestone and shale	Sandstone and shale
Sonic-neutron cross plot	Limestone, shale and sandstone	Shale, sandstone and limestone
M-N cross plot	Limestone	Sandstone and shale
MID plot	Limestone and shale	Sandstone and shale

#### Density-resistivity/neutron-resistivity overlays

**Fig. 5:**
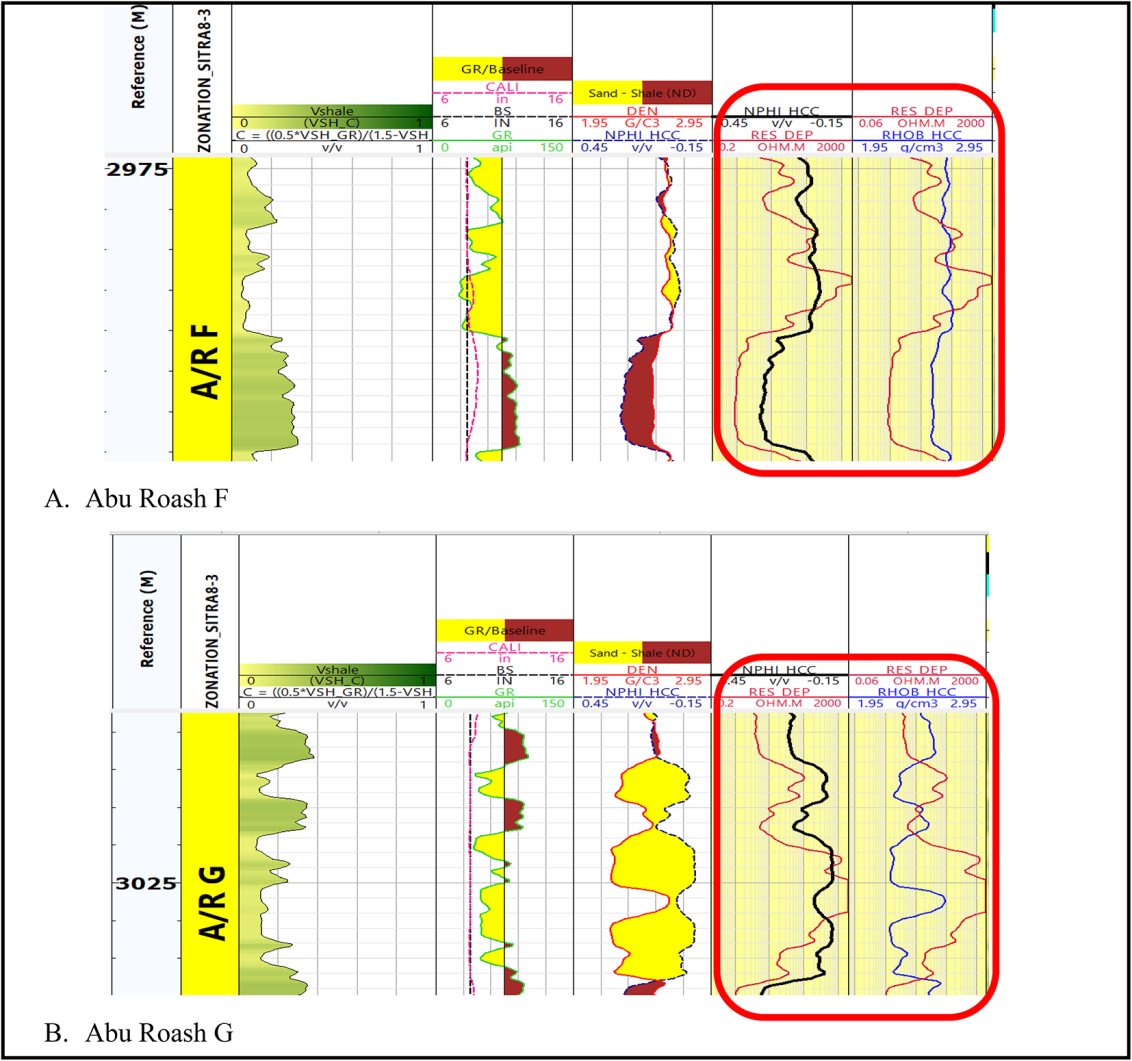
Highlighted the density-resistivity overlay and neutron-resistivity overlay in (**A**) A/R F formation shows complete resemblance in carbonate rock (**B**) A/R G formation shows lack in similarity in clastic sedimentary.

The density-resistivity overlay and neutron-resistivity overlay are utilized to distinguish non-clastic rocks from clastic sedimentary formations. The similarity between the two overlays is ascribed to their carbonate composition, whereas the dissimilarity is due to their clastic character. Consequently, it may be stated that the density-resistivity overlay and neutron-resistivity overlay are preferred utilized for non-clastic rocks rather than clastic sedimentary formations^10,13,32^ (Fig. 5). The presence of the organic matter in A/R F results in diminished readings for density, whilst the resistivity curve is characterized by elevated values^10,13,32^ (Fig. 5A).

#### Density–neutron/sonic-neutron cross plots

**Fig. 6:**
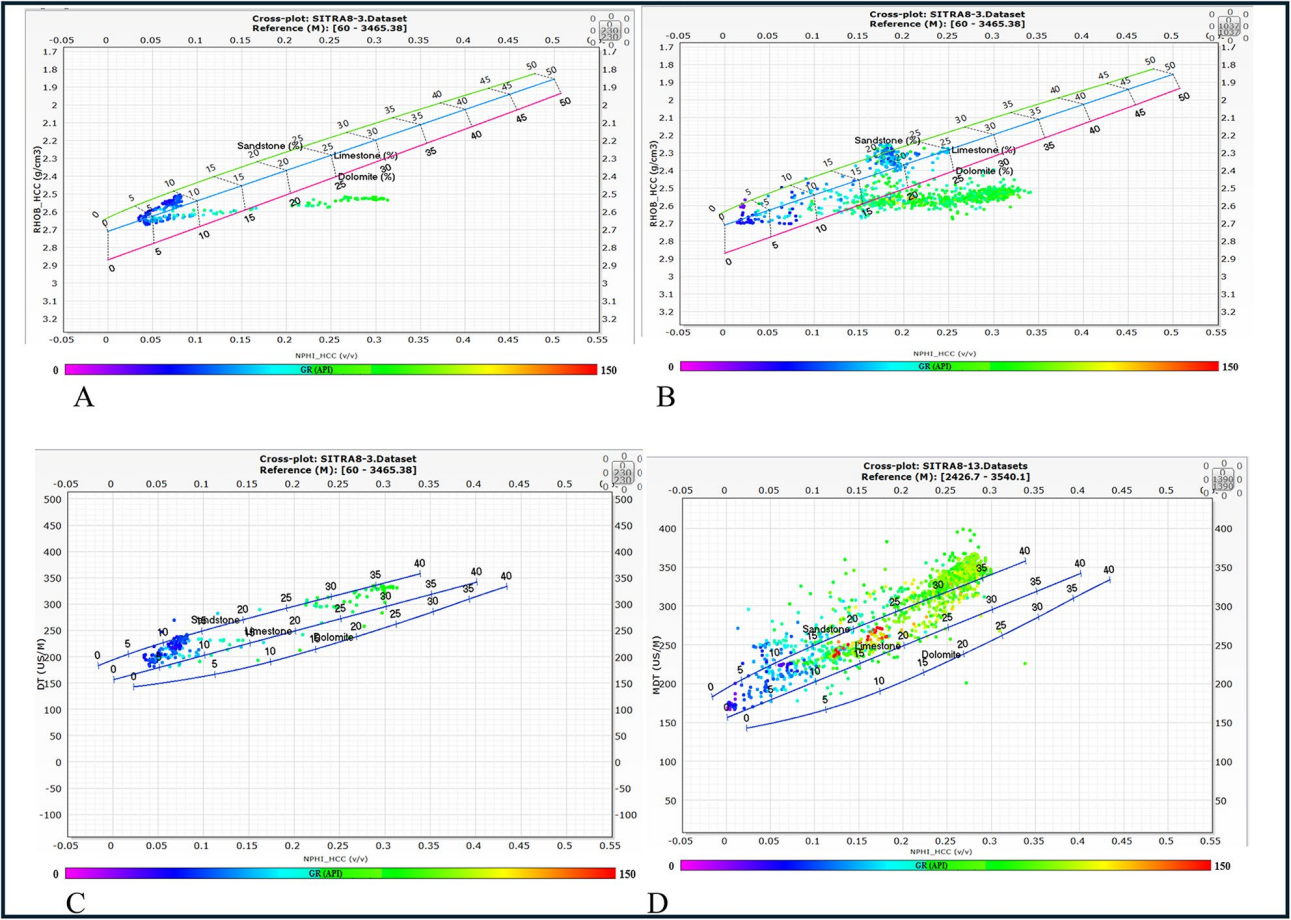
(**A**) Density-Neutron crossplot for A/R F, (**B**) Density-Neutron crossplot for AR/G, (**C**) Sonic-Neutron crossplot for A/R F, (**D**) Sonic-Neutron crossplot for AR/G.

Density–Neutron/Sonic-Neutron cross plots were developed using logs data for AR/F & AR/G formations to determine the lithological composition. Figure 6 showed that the dominating lithology in AR/F is limestone and shale, while the dominating lithology in AR/G is sandstone and shale, the color code represents the GR values which is an indication for the shale content.

#### M–N plot and MID plot

**Fig. 7:**
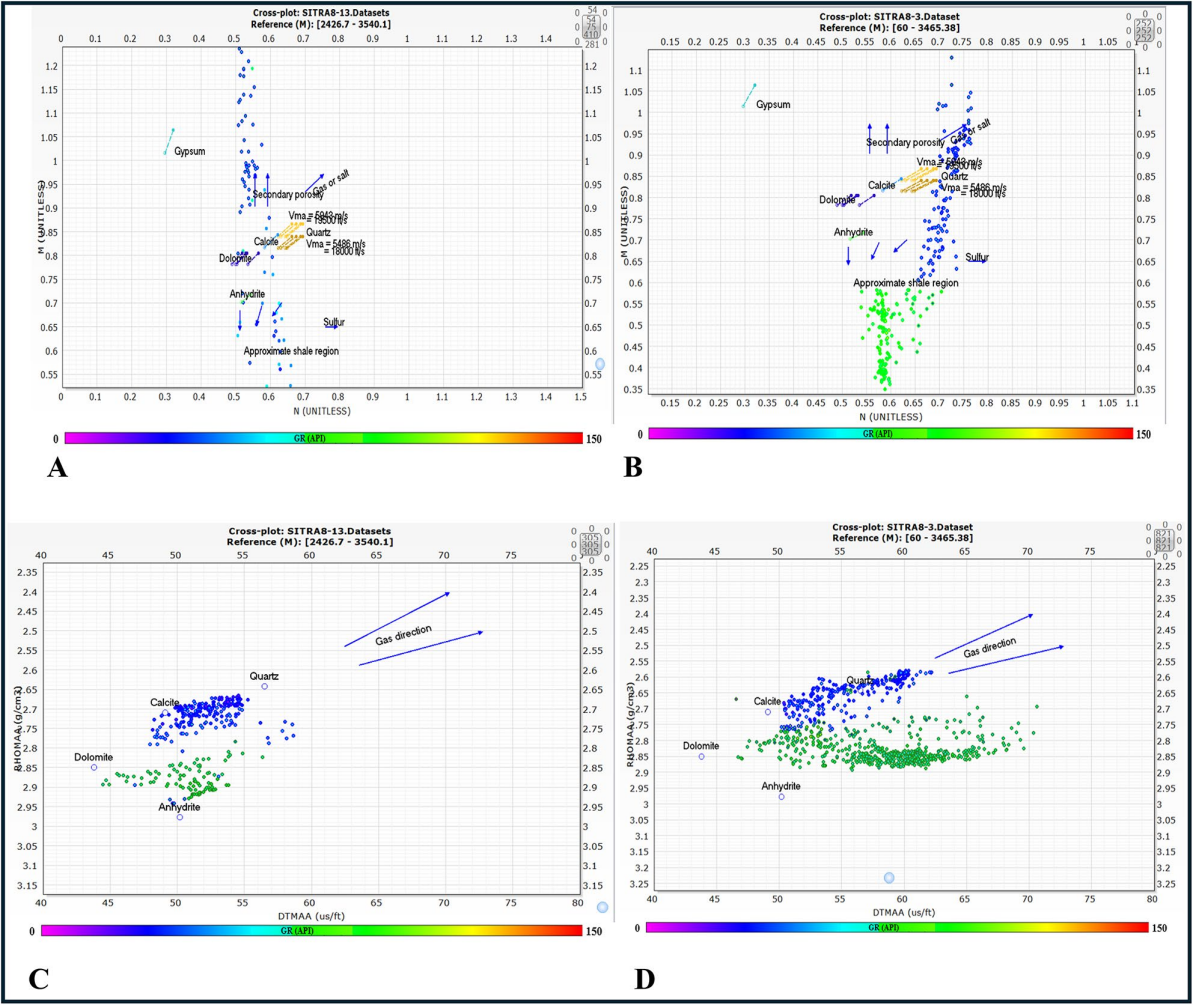
(**A**) M-N Cross plot of AR/F, (**B**) M-N Cross plot of AR/G, (**C**) MID cross plot of A/R F, (**D**) MID cross plot of AR/G.

The M–N plot was utilized for matrix identification by means of calculating M and N values. The value of M was determined using the sonic and density logs. The value of N was determined using the neutron and density records. The M and N values were calculated using the following formulae. The matrix type can be determined by plotting the intersection point of the M and N values^33^.

1 $$\:M=\frac{{\Delta\:}T\:f-{\Delta\:}T\:log\:}{\rho\:b-\rho\:f}\times\:0.01\:$$

2 $$\:N=\frac{\varnothing\:Nf-\varnothing\:Nlog}{\rho\:b-\rho\:f}$$

where ∆Tf = the transit time of the fluid, ∆Tlog = the transit time of the formation, ρf = the density of the fluid, ρb = the formation bulk density. ∅Nf = the neutron porosity for fluid, ∅N-log = the neutron porosity of the formation. In regard to MID plot, the values for the apparent grain density (ρma) and apparent matrix transit time (Δtma) will be inputted into the matrix identification MID plot^34^ (Fig. 7). The results indicated that AR/F composed of calcite and shale while, AR/G composed of sandstone and shale.

### Hydrocarbon indicators

Techniques identify potential hydrocarbon-rich layers. In this case study, we utilized the following^29^.

1. FR–Ro overlays^35^.
2. The neutron–density overlay.
3. The resistivity–porosity overlay technique^32^.
4. Bulk volume of water (BVW)^36^.

#### FR–Ro overlays

An important parameter in formation evaluation is the formation resistivity factor of a porous media It is defined as the ratio of the medium’s resistivity to that of the saturating fluid when the medium is saturated with the conducting fluid as follows^35^.

3 $${\text{FR}}={\text{Ro}}/{\text{Rw}}$$

FR = Formation resistivity factor, Ro = Resistivity of water saturated S.S. (Ω m), RW = is the resistivity of water in formation (Ω m).

**Fig. 8:**
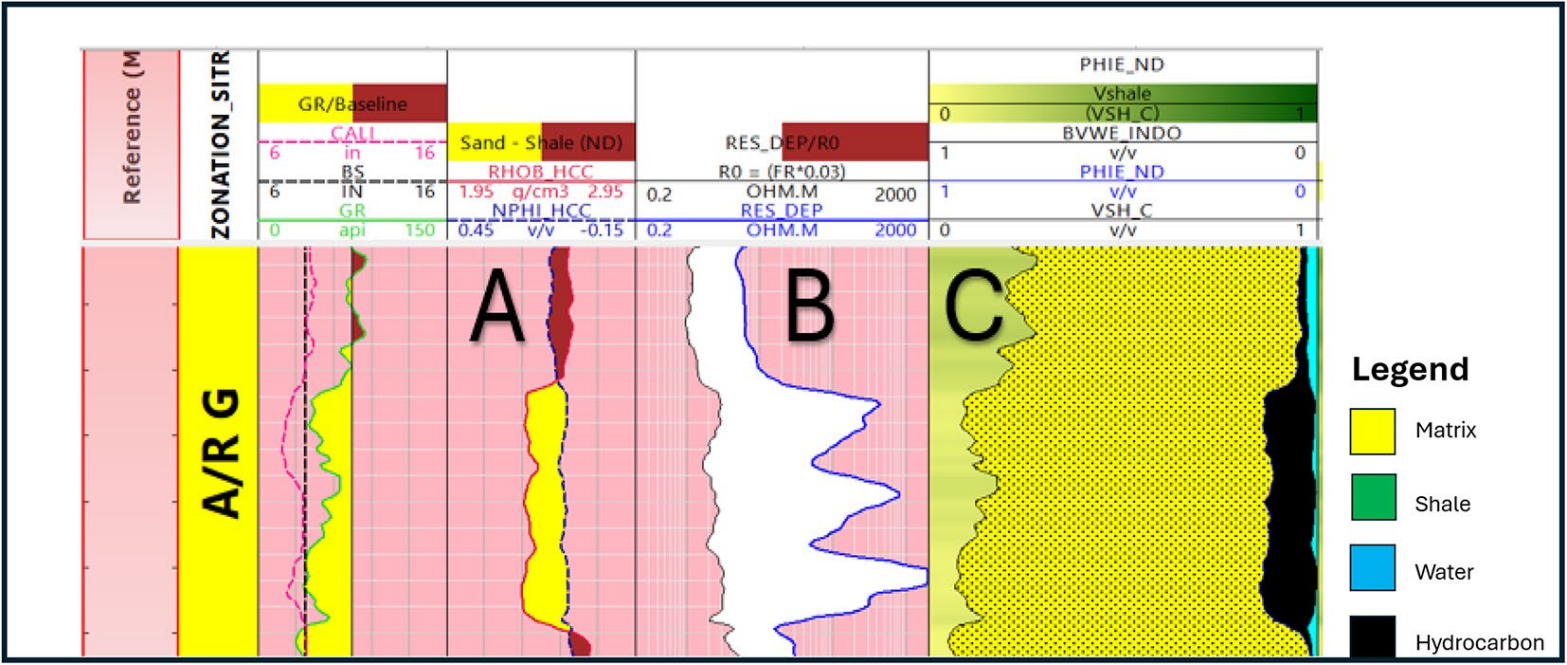
(**A**) The neutron–density overlay, (**B**) FR-Ro overlay & (**C**) bulk volume of water (HC Bearing Zone) of upper AR/G.

**Fig. 9:**
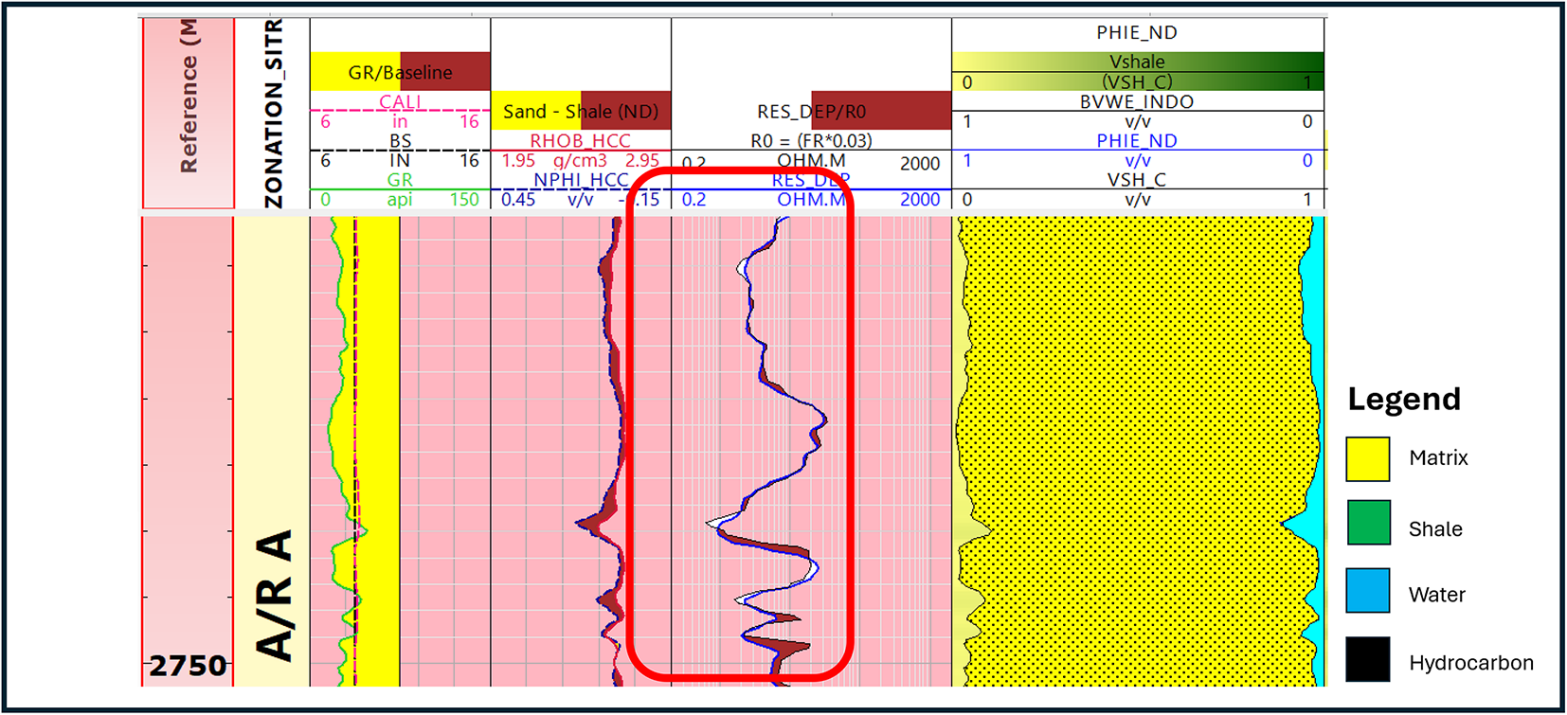
FR-Ro overlay (water bearing zone).

Within hydrocarbon-rich areas, there is a separation between R0 and FR (Figs. 8, 9).

#### The neutron–density overlay

The presence of hydrocarbons can be identified by analyzing the separation between the density and neutron logs (Fig. 8). Gases can cause significant separation, while oil can cause minor separation.

#### The resistivity–porosity overlay technique (Δ log R)

**Fig. 10:**
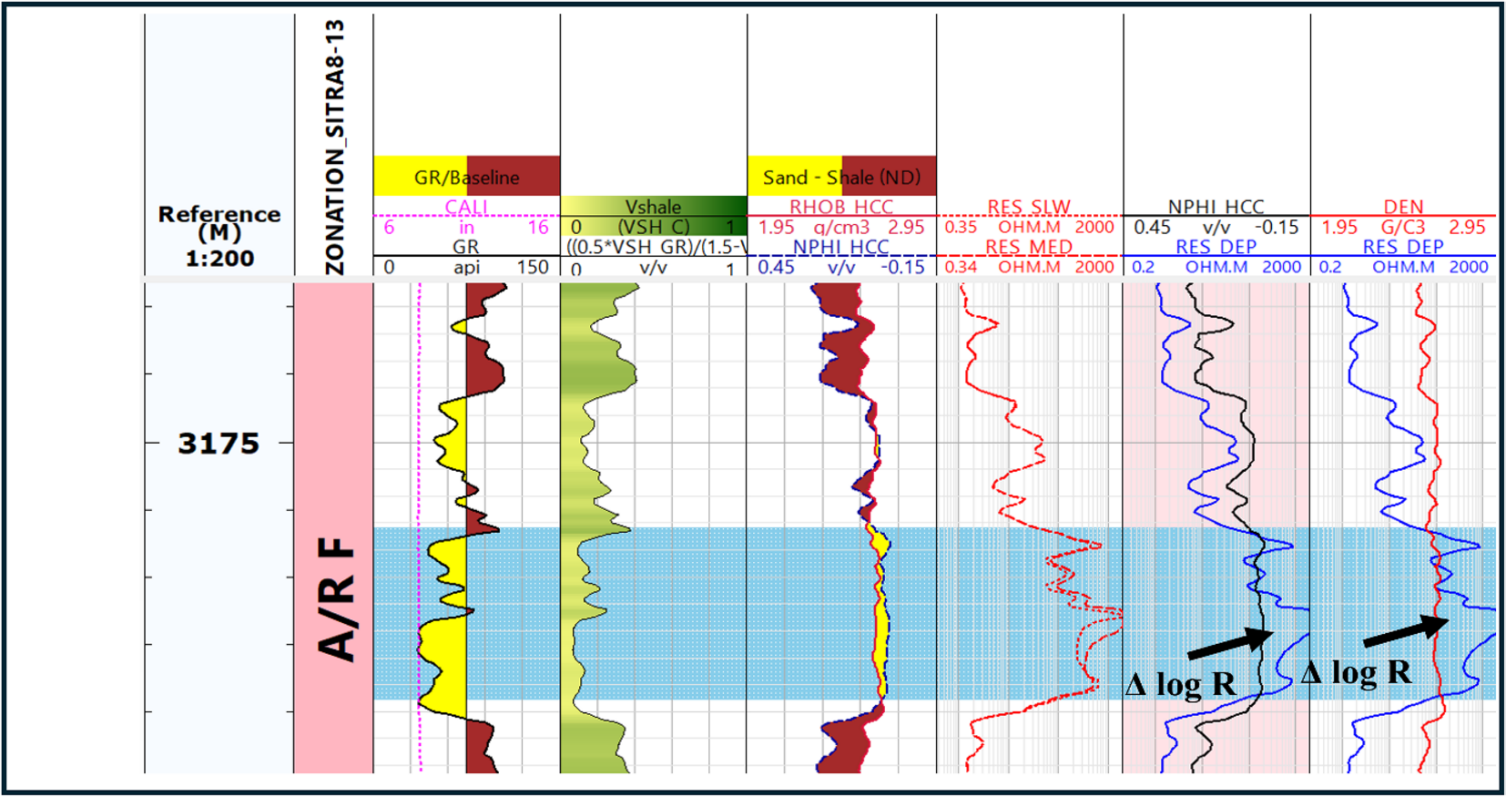
The density-resistivity overlay and neutron-resistivity overlay in A/R F (highlighted in blue) providing proof of the presence of organic matter by demonstrating the, agnitude of separation (Δ log R).

In organic-rich rocks or hydrocarbon reservoirs, the resistivity–porosity (Neutron/Denisty) curves diverge. This separation, occurring in organic-rich intervals, creates a gap due to the presence of organic matter (Fig. 10), resulting in low density, while the resistivity curve exhibits elevated values in reaction to organic matter^10,13,32^.

#### Bulk volume of water (BVW)

The utilization of the available tools to plot porosity and BVW simultaneously provides a straightforward and effective indicator for zones containing hydrocarbons and water (Fig. 8)^29,36^.

4 $${\text{BVW}}={\text{ PHI eff }}*{\text{ Sw}}$$

PHI eff = effective porosity, Sw = Water Saturation.

### Pickett plot and rw determination

**Fig. 11:**
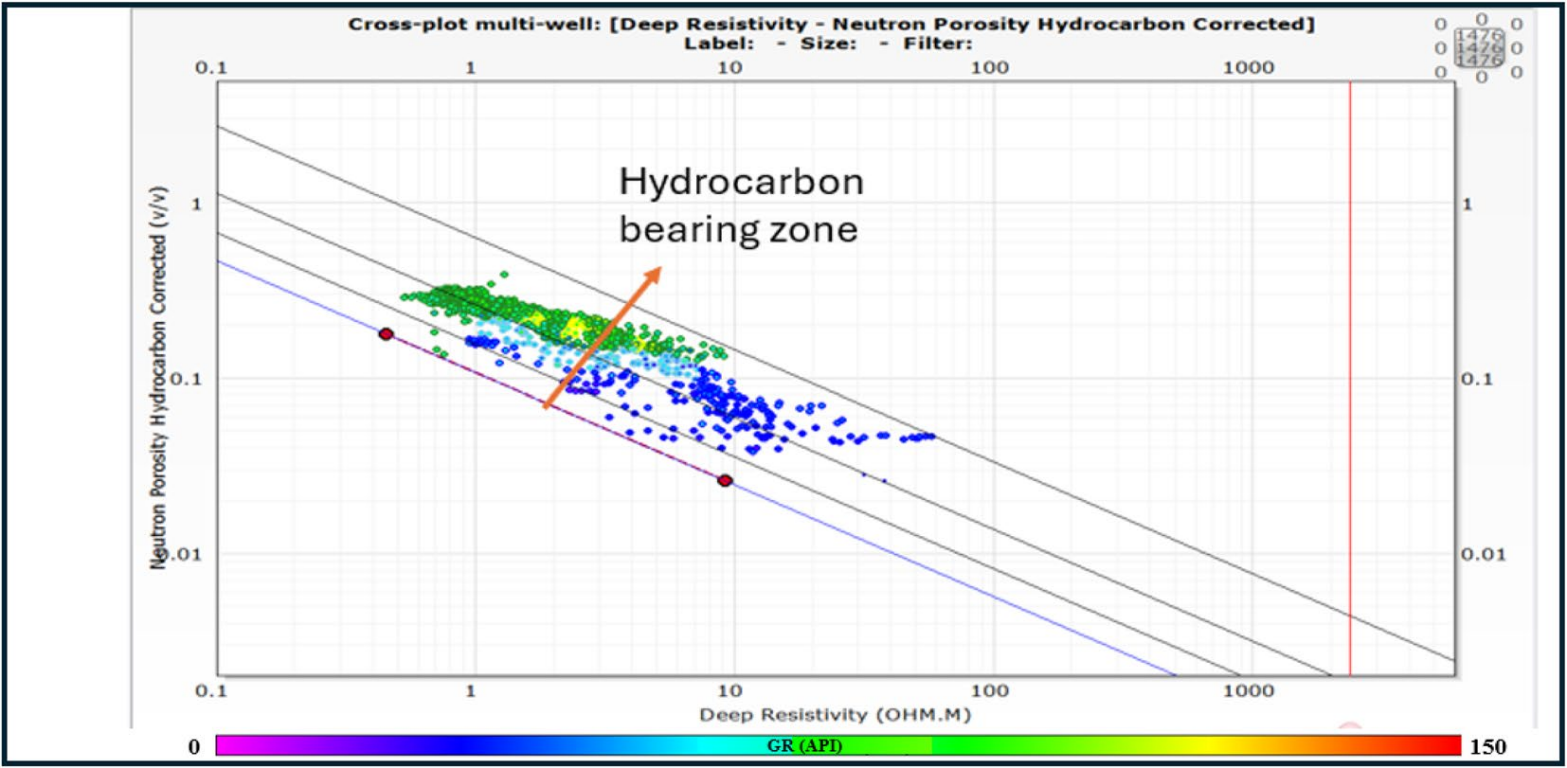
Pickett plot.

Pickett plot is a method that provides a graphical solution to Archie’s equations^37^ to estimate water resistivity value (Rw), Cementation Factor (M), and water saturation (Sw), Eq. (5) by plotting formation resistivities (X-Axis) with corresponding Neutron porosities (Y-Axis) taken from logs on a log-log scale^25,27,29,38^. The calculation gave the proportionality factor ( = 1), cementation component (m = 1.6), the saturation coefficient (*n* = 2) and the resistivity of water in formation (Rw = 0.032) from Pickett plot (Fig. 11).

5 $$\:{\left(Sw\right)}^{n}=\sqrt{\frac{a\text{*}Rw}{{{\varnothing}}^{m}\text{*}Rt}}\:\text{A}\text{r}\text{c}\text{h}\text{i}\text{e}\:\text{E}\text{q}\text{u}\text{a}\text{t}\text{i}\text{o}\text{n}$$

Sw = Water saturation, a = proportionality factor (in range of 0.5 to 1.4), m = cementation component (in rang of 1.25 to 2.95), n = The saturation coefficient, which is commonly put as 2, R = The resistivity of water in formation.

### Petrophysical parameters (vsh, φeff, SW & BVW)

#### Shale volume

Shale volume refers to the proportion of shale in a formation, which is calculated by dividing the quantity of clay content by the overall volume of the formation. The computation of shale volume, based on the gamma-ray log, employs a linear technique^39^ using the Eq. (6)^40^.

Accurate evaluation of shale volume is crucial since it has a significant impact on many petrophysical properties, including effective porosity and water saturation^5^. The calculated Vsh value leads to an exaggerated estimation of the shale volume, resulting in a negative assessment of the reservoir’s quality. Hence, the ultimate value should be rectified by utilizing one of the techniques presented by^27,29,41^.

6 $$\:\text{V}\text{s}\text{h}\:=\:\frac{GRlog-GR\:min}{GRmax-GR\:min}$$

7 $$\:\text{V}\text{s}\text{h}\:\text{s}\text{t}\text{e}\text{i}\text{b}\text{e}\text{r}\:=\:\frac{0.5\text{*}Vsh}{1.5-Vsh}$$

Vsh = volume of shale, GRLog = gamma ray reading, GRmax = maximum gamma ray (shale), GRmin = minimum gamma ray (clean sand or carbonate).

#### Porosity calculations

The oil industry classifies porosity as follows^29^.

**Fig. 12:**
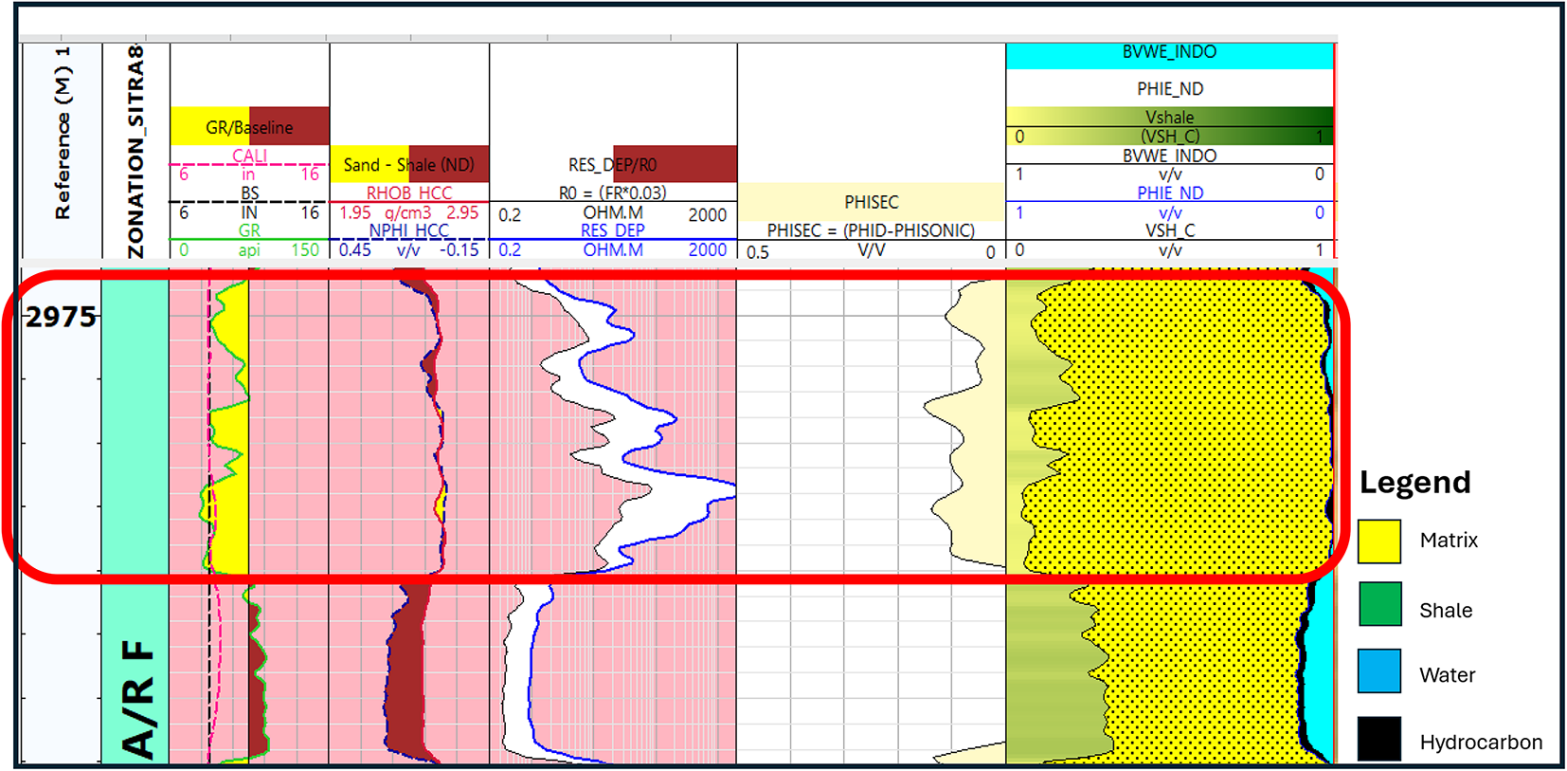
Highlighted zone of secondary porosity in A/R F.

- Total porosity: The proportion of the bulk volume to the total pore space within a rock.
  8 $$\:\text{P}\text{H}\text{I}\:\text{t}\text{o}\text{t}\text{a}\text{l}\:=\:\frac{PHI\:N+\:PHI\:D\:}{2}$$
  9 $$\:\text{P}\text{H}\text{I}\text{D}\:=\:\frac{\rho\:ma-\:\rho\:\:b}{\rho\:ma-\:\rho\:f}$$
  PHIT = Total Porosity, PHIN = Porosity from Neutron log (amount of hydrogen), PHID = Porosity from Denisty log Eq. (2), $\:\rho\:$ma = Matrix Density, $\:\rho\:$b = bulk density (density log), $\:\rho\:$f = fluid density (1.1 salt mud, 1 fresh mud, 0.7 gas).
- Effective porosity: The proportion of the overall pore volume through which fluid permeates the entire volume of the rock.
  10 $${\text{PHIeff }}={\text{ PHIN }} - ({\text{VshSteiber}} * {\text{PHIsh}})$$
  PHIeff = Effective Porosity, PHIN = Porosity from Neutron log (amount of hydrogen), Vsh Steiber = corrected shale volume, PHIsh = shale porosity.
- Secondary porosity must also be determined, as our study includes the examination of AR/F (carbonate rock) (Fig. 12). secondary porosity is the porosity caused by post-depositional alteration of the initialsediment^1^. It is one of the defining characteristics of carbonate reservoirs and it could be calculated as follows:
  11 $${\text{Secondary porosity}}\,=\,{\text{PHIT}}\, - \,{\text{PIHS}}$$
  12 $$\:=\text{P}\text{H}\text{I}\text{T}\:-\left(\frac{\varDelta\:t\text{log}-\:\varDelta\:t\:ma}{\varDelta\:t\:fl-\:\varDelta\:t\:ma}\right)$$
  PHIT = Total Porosity, PHIS = sonic porosity, ∆t log = interval transit time of the formation (from the sonic log), ∆t ma = matrix interval transit time, ∆fl = fluid interval transit time (185 μs/ft for saltwater-based mud and 189 μs/ft for freshwater- based mud).

#### Water saturation (SW) and bulk volume of water (BVW)

To figure out how promising hydrocarbon-bearing formations (1-SW) are, petrophysical parameters like porosity (θ), volume of shale (Vsh), and water saturation (Sw) are often used. The simple conductivity model of Archie’s equation can be used to figure out water saturation in clean formations. However, in shaley sand reservoirs, the clay minerals add extra conductivity, which could change the estimated water saturation by using the Indonesia equation^42^. Water saturation is based on deep resistivity (Rt), while shale resistivity (Rsh) is determined by logging versus pure shale zones^25,43,44^.

13 $$\:\frac{1}{\sqrt{Rt}}\:=\:\left[\sqrt{\frac{{{\varnothing}}_{m}}{aRw}}+\frac{{Vsh}^{\left(\frac{1-Vsh}{2}\right)}}{\sqrt{Rsh}}\right]\:{SW}^{n}$$

Sw = Water saturation, a = proportionality factor (in range of 0.5 to 1.4), m = cementation component (in rang of 1.25 to 2.95), n = The saturation coefficient, which is commonly put as 2, R = The resistivity of water in formation, Rt = True deep resistivity in un-invaded zone, Vsh = shale volume, $\:{\varnothing}$ = effective Porosity, Rsh = Resistivity of shale.

The bulk water volume (BVW) was then determined by multiplying the obtained water saturation water (Sw) with its equivalent porosity by the equation of^45^.

14 $$BVW=SW*\phi eff$$

BVW = Bulk volume f water, SW = Water saturation, ∅ = Effective porosity.

**Fig. 13:**
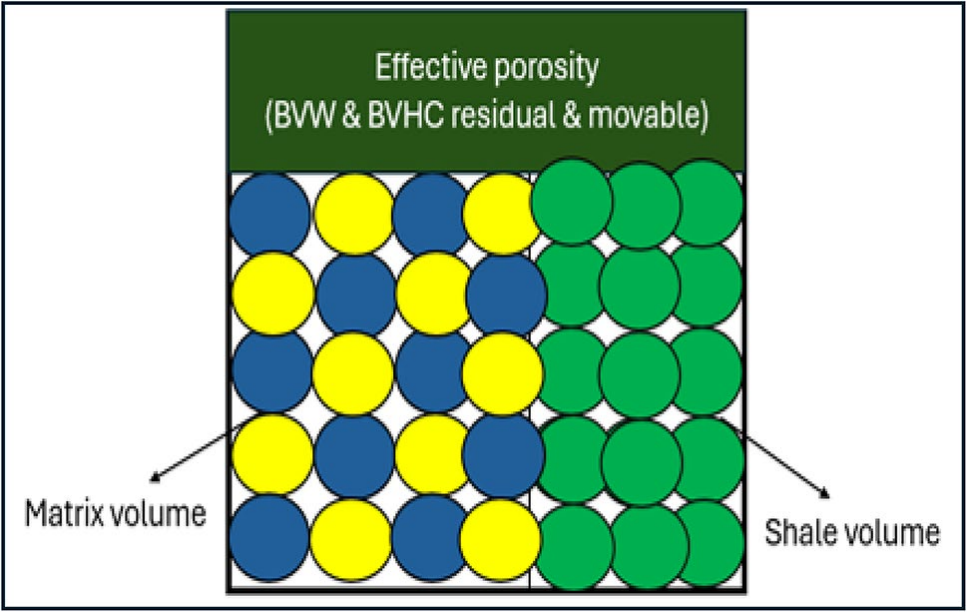
Formation rock model.

**Fig. 14:**
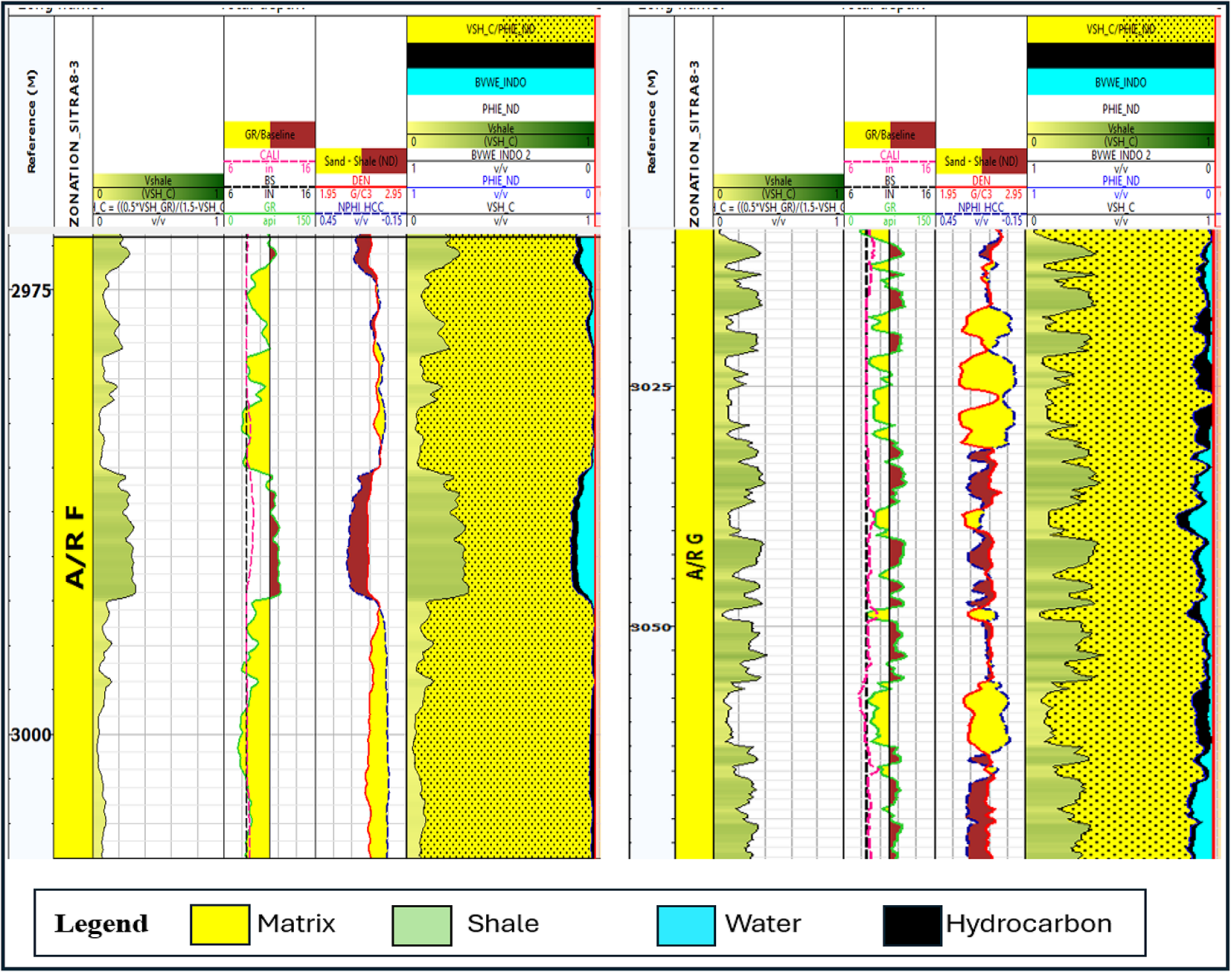
Formation rock model of A/R F well (SITRA8-03) & formation rock model of A/R G well (SITRA8-03).

Bulk volume water represents the proportion of water in a unit volume of reservoir rock. Once the volume of shale, effective porosity, and bulk volume of water have been computed, it is possible to develop a bulk volume rock model (Figs. 13, 14) that provides information regarding the percentage of matrix volume in addition to the volume of shale and the percentage of hydrocarbon saturation^27^.

Separating the movable hydrocarbons (found in the pore spaces of the reservoir rock and can be extracted using artificial lift methods or natural reservoir pressure) from the residual hydrocarbons (which are those that are still trapped in the reservoir rock) could be obtained by using a movable oil plot (MOP). We applied three logs (1) Formation factor log – F. (2) True formation Resistivity divided by formation water resistivity – Rt/Rw. (3) Invaded zone Resistivity divided by mud filtrate Resistivity – Rxo / Rmf^25,27–29,33^.

**Fig. 15:**
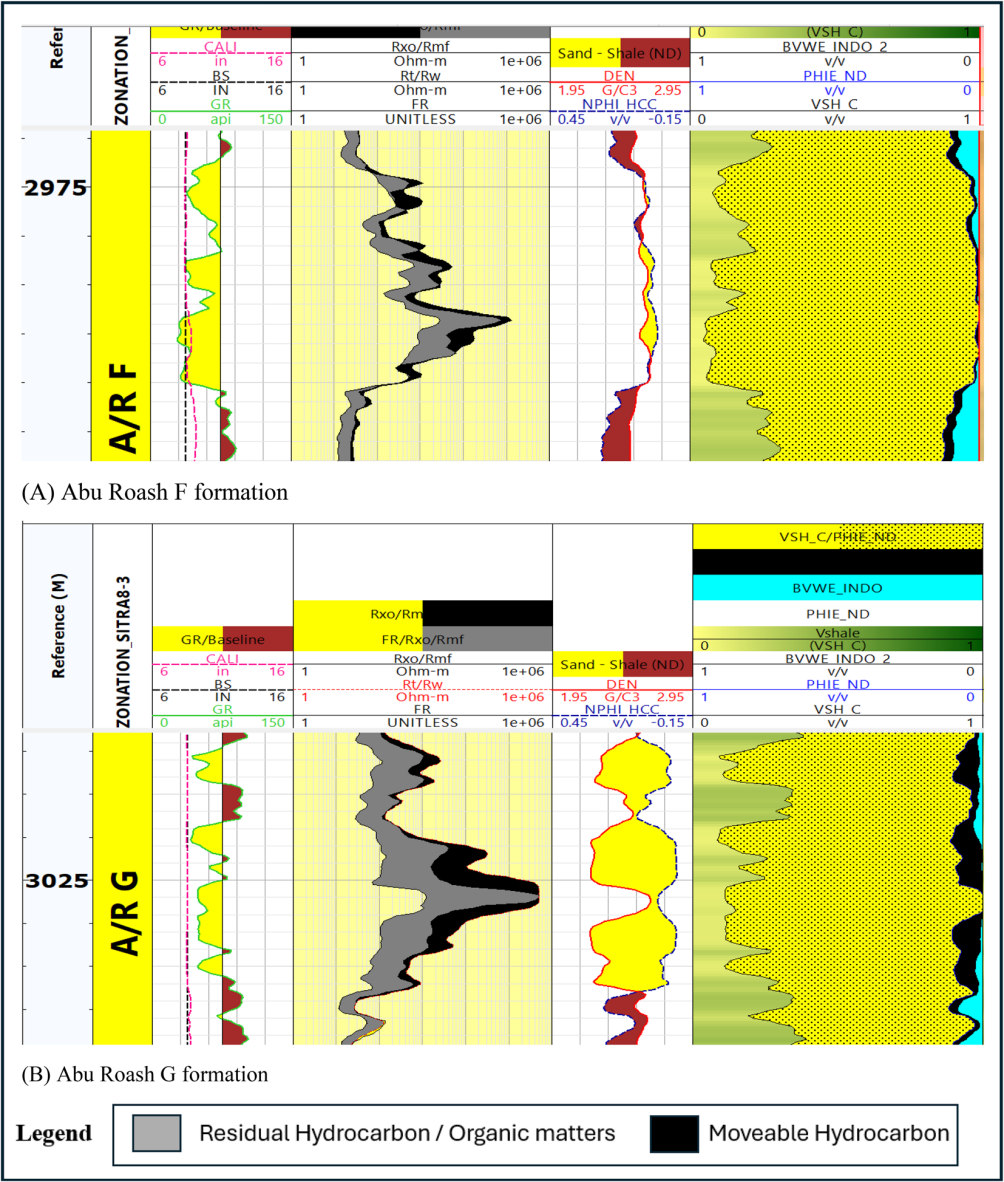
Moveable oil plot (MOP) (**A**) Abu Roash F formation, (**B**) Abu Roash G formation.

Figure 15 declares the separation between $\:F$ and (Rxo / Rmf ) which reveal whether residual hydrocarbon is present, also (Rxo / Rmf ) and (Rt / Rw) curve separation, indicate the existence of movable hydrocarbons will be visible.

The organic matter is taken into consideration as a reservoir’s residual hydrocarbon source. When organic-rich does not reach thermal maturation as in our case in A/R F in Fig. 15.

### Core analysis

**Fig. 16:**
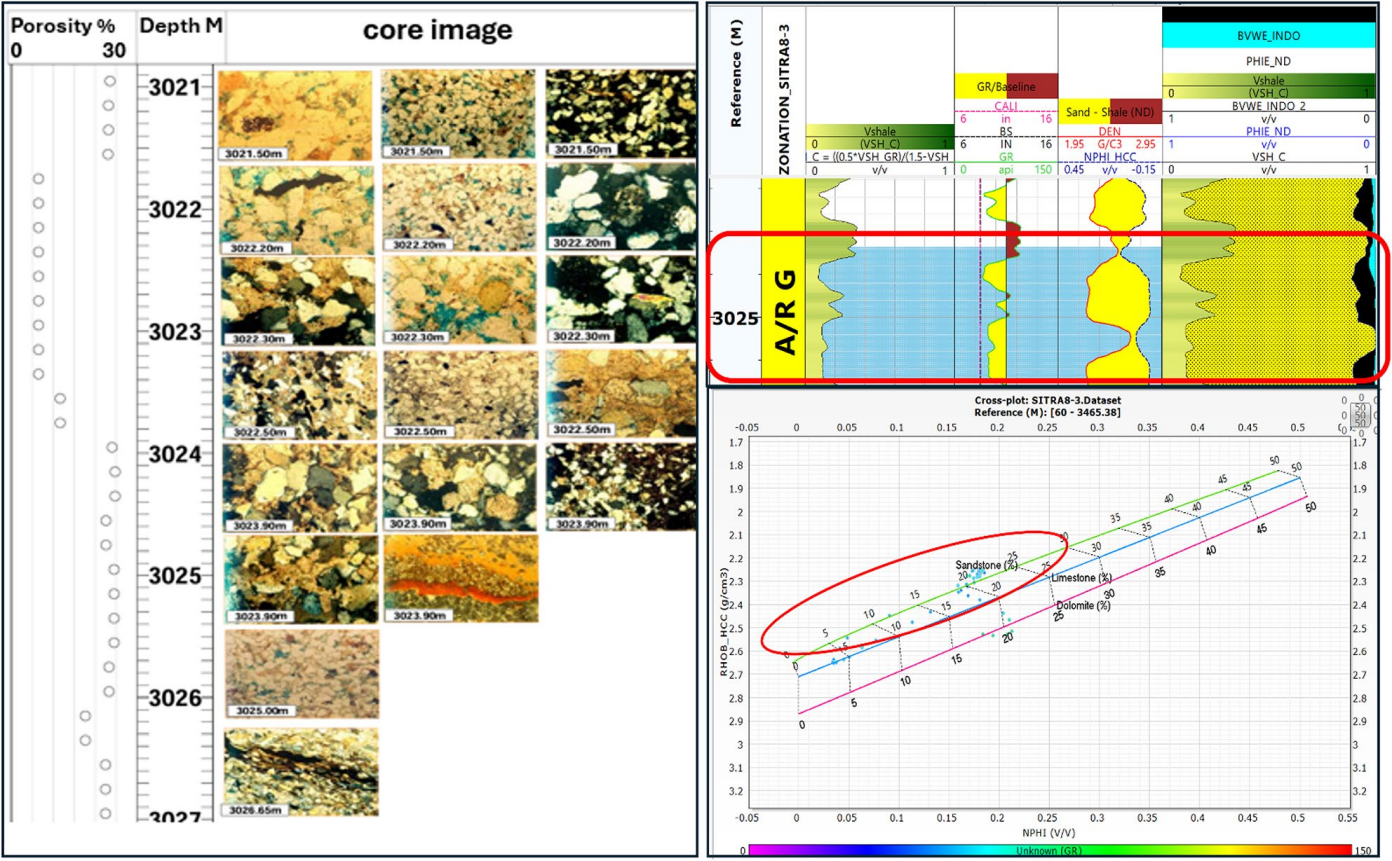
(**A**) Petrographic images of core samples, (**B**) Petrophysical analysis, (**C**) Density–Neutron cross-plot in well SITRA8-03 belonging to the interval (3021–3027 m) upper AR/G formation.

**Fig. 17:**
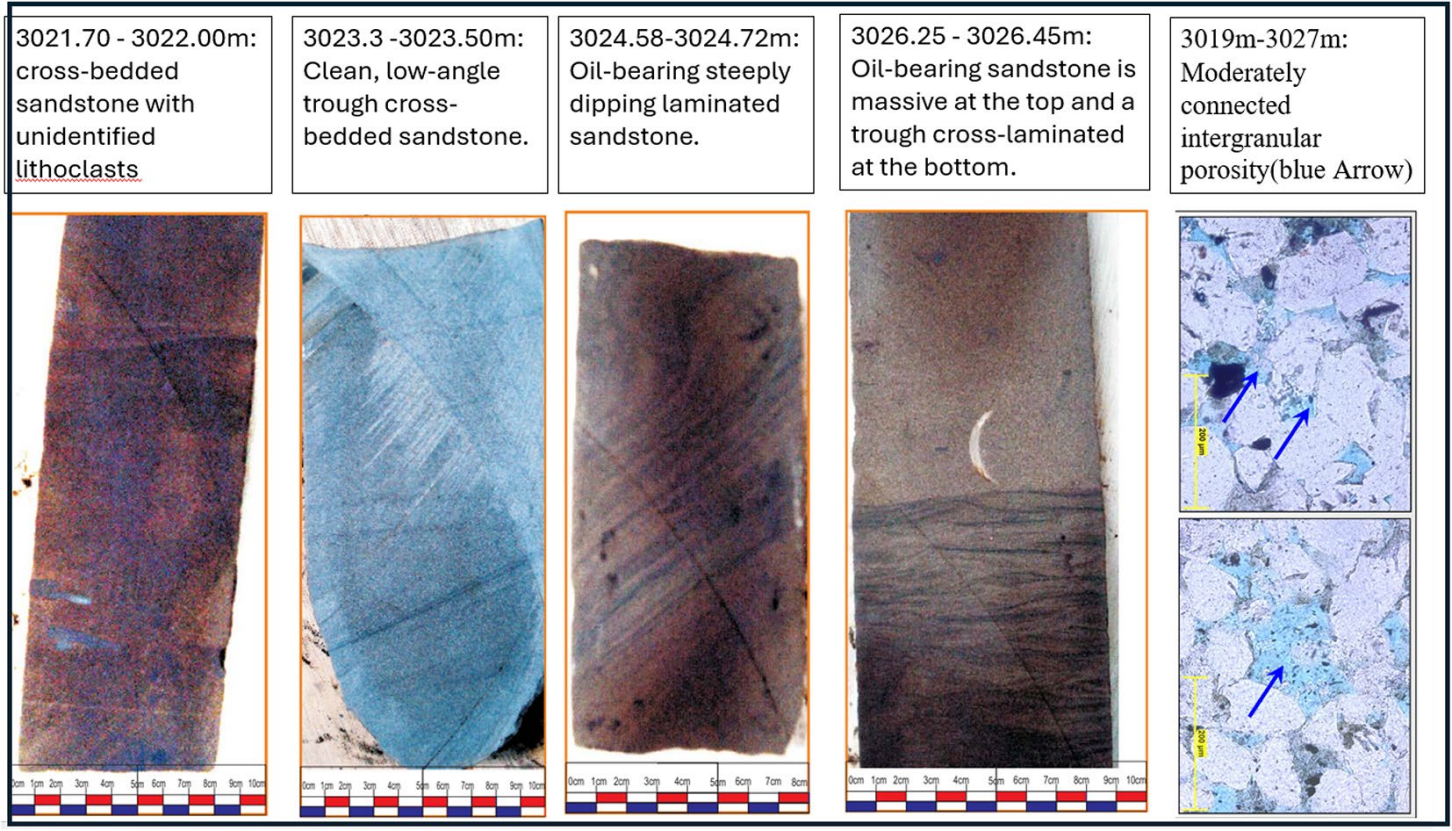
Core Slabs of the interval (3021–3027 m) show the lithofacies type with moderately connected intergranular porosity (blue Arrow) in Well SITRA8-03 Of upper A/R G.

**Fig. 18:**
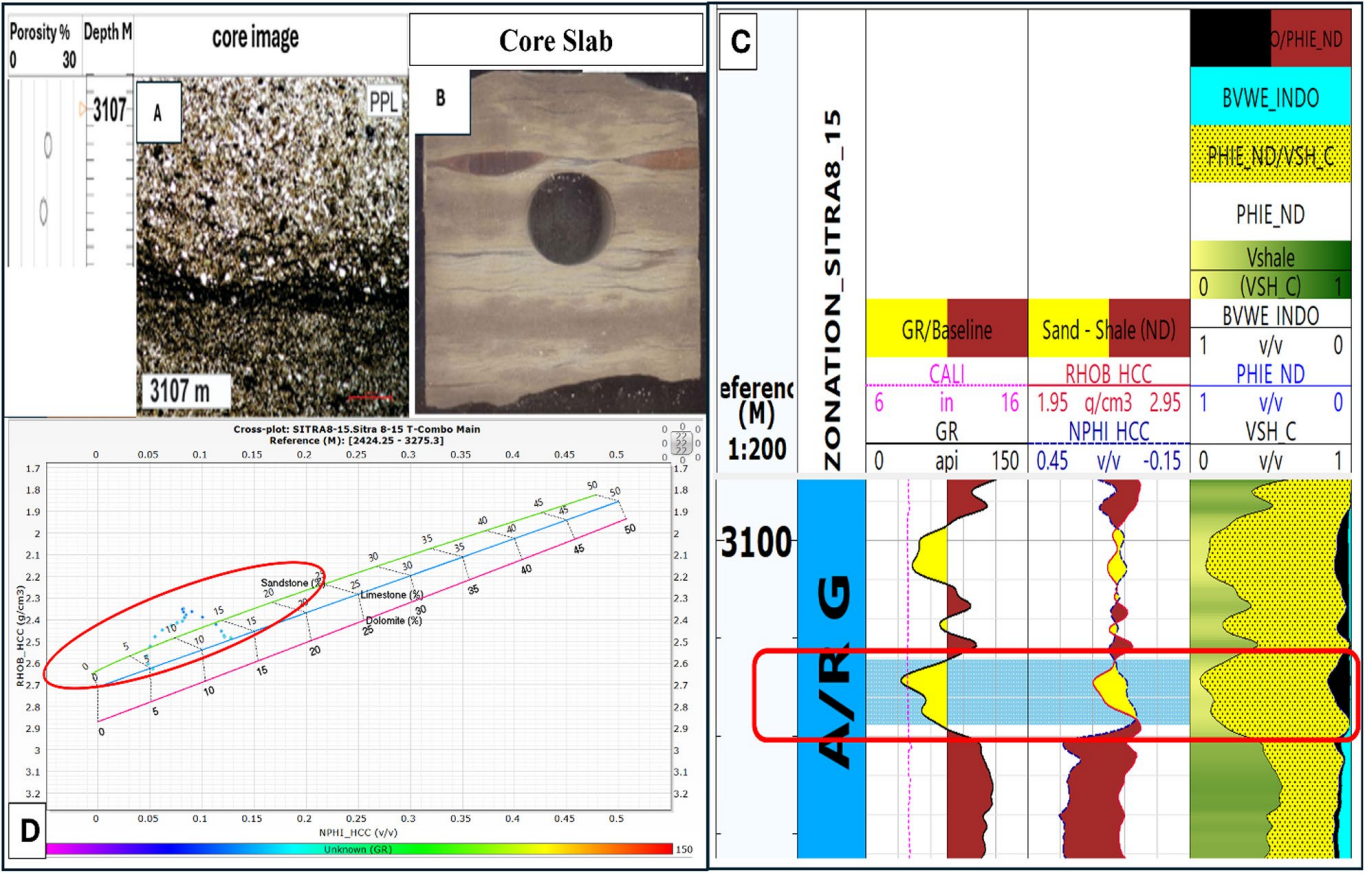
Fine-grained laminated sandstone at depth 3107 m in well sitra8-15 (**A**) petrographic image, (**B**) core slab, (**C**) petrophysical analysis, (**D**) density–neutron cross-plot.

The logs data supported with core samples are available only at the upper part of A/RG formation provided the results of the lithologic description, lithofacies analysis, paleoenvironmental interpretation petrographic analysis, and porosity of AR/G formation. Table 2 presents these results in Well SITRA8-03 and SITRA8-15. In Sitra8-03 There is a significant sandstone interval (3019–3027 m) that draws attention to it in our research. Figure 16 correlates well logs, Density–Neutron cross-plot, and core samples and they ensure each other. This interval is characterized by massive sandstone and cross-bedded Sandstone lithofacies type with Moderately connected intergranular porosity(blue) (Fig. 17). Whereas shale dominates the lithology in well SITRA8-15, The microscopically visible porosity is generally low to nil, and Good open microporosity was only revealed at 3107 m where a layer of fine-grained laminated sandstone is present (Fig. 18). Well logs analysis and Density–Neutron cross-plot address the presence of the sandstone which correlate with the core sample and core slab obtained from Badr El-Din company.

**Table 2:** Core analysis of SITRA8-03 and SITRA8-15 samples.

Well	SITRA8-03	SITRA8-15
Samples interval	27.15 m thick interval from depth 3012.4 m to depth 3039.55 m	27.35 m thick interval from depth 3089 m to depth 3116.35 m
Lithology	Sandstone interbedded with shale	Shale interbedded with sandstone
Depositional environment	Possibly Tidally-dominated environment	Nearshore marine environment for clastic deposition
Paleontological analysis (Fossils)	foraminifera and ostracoda	Common foraminifera
Dominant Lithofacies analysis	Wavy laminated sandstone Cross bedded sand stone Massive sandstone Laminated shale	Laminated shale Bioturbated and flat laminated sandstone
Porosity values	Range between 4 and 14% Pore interconnectivity ranges from poor to moderate-good	Generally low to nil

### Area interpretation

#### Mapping

**Fig. 19:**
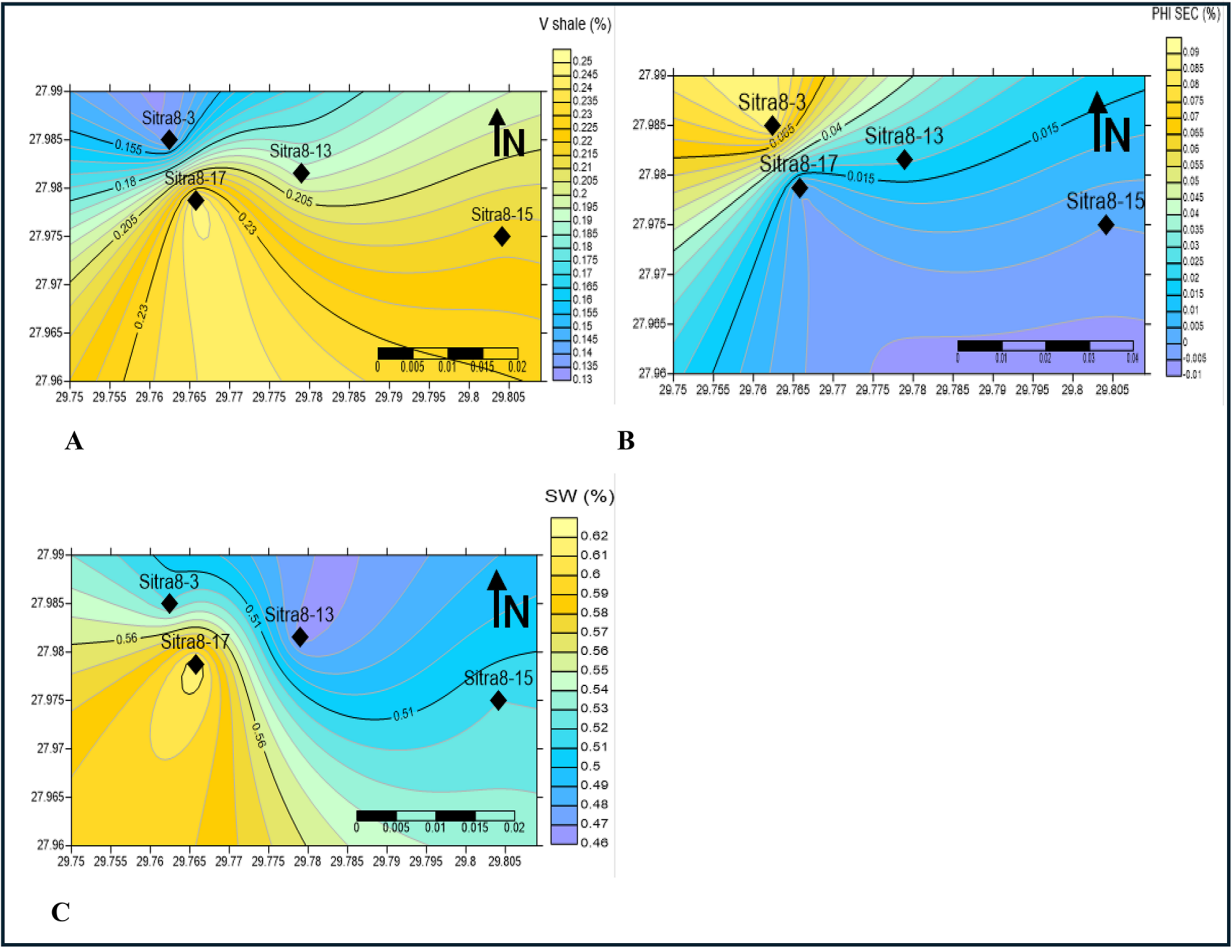
Contour map by surfer 13 software (**A**) volume of shale, (**B**) secondary porosity, (**C**) water saturation of A/R F formation.

**Fig. 20:**
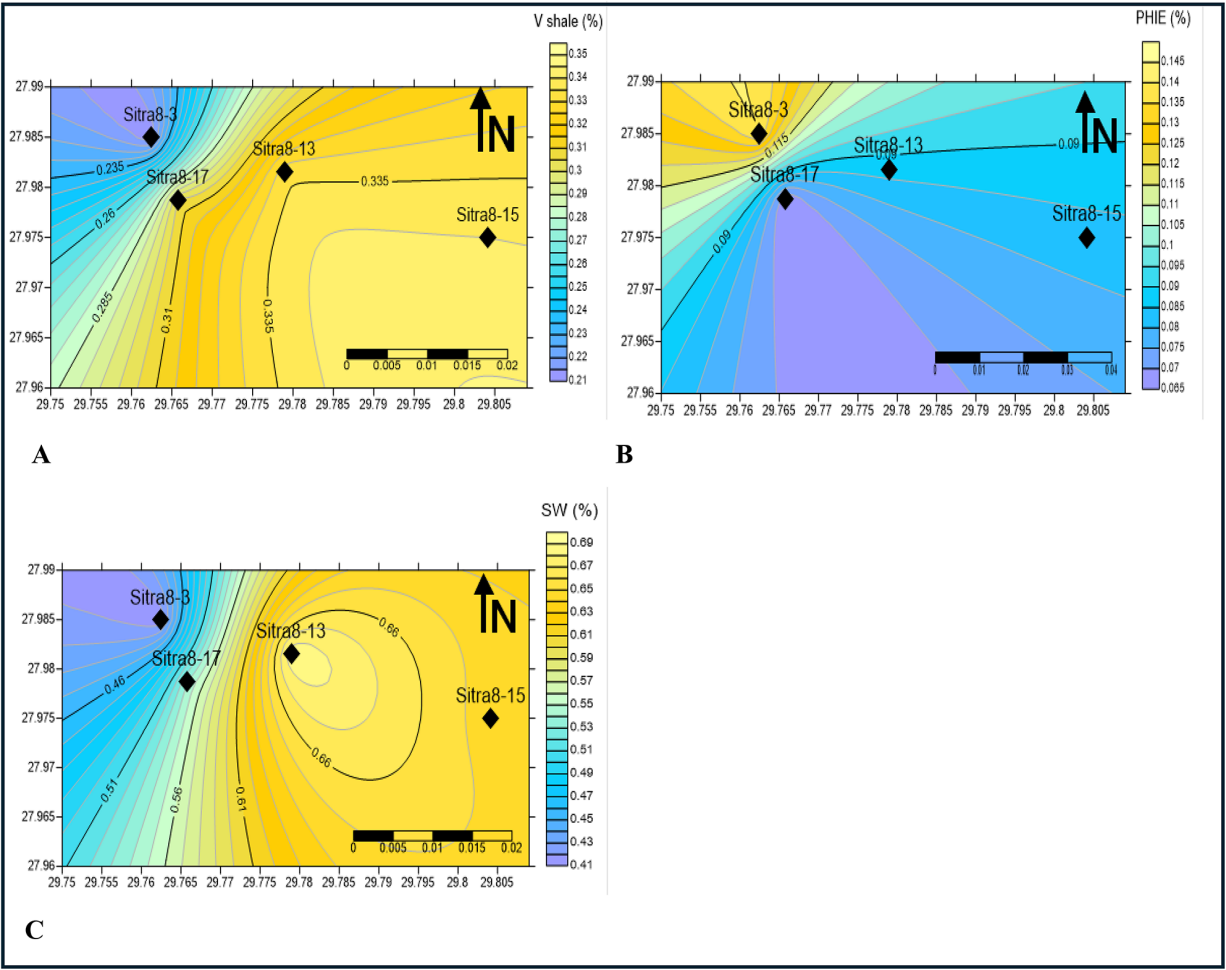
Contour map by surfer 13 software (**A**) volume of shale, (**B**) effective porosity, (**C**) water saturation of A/R G formation.

Cartographic representations of the reservoir’s shale content, Average porosity, and water saturation are generated via surfer 13 software in order to assess the spatial distribution of hydrocarbon potential within the designated study area. As shown in Figs. 19 and 20, the estimated distribution of petrophysical parameters for different potential zones.

Concerning AR/F formation (Fig. 19) (unconventional carbonate reservoir)^14,46^, the volume of shale diminishes in the northwest direction, while secondary porosity due to the presence of fractures increases correspondingly. Water saturation declines towards the north; thus, drilling development wells in the northwest direction is highly recommended.

##### Depositional environment

The Abu Roash F Member in the Abu El Gharadig Basin was deposited in calm marine settings and inner to middle platform environment^47–52^. These environments are defined by:

- Calm marine settings: These regions generally consist of deeper waters with minimal energy, facilitating the deposition of fine-grained sediments and organic-rich strata.
- Inner to middle platform: These habitats exist on the continental shelf, where carbonate sediments build, creating possible reservoir rocks. These settings are critical for hydrocarbon exploration because they can support high-quality reservoir and source rocks as a result of organic material accumulation and secondary porosity development due to marine transgression when the sea advances over the land^47–52^.

Contour maps (Fig. 19) demonstrate the depositional environment of A/R F as the expansion of the shale volume is in the south and southeast directions suggesting a result of fine-grained clay content, poor sorting, and poor porosity. Additionally, the secondary porosity in A/R F increases towards the northwest, indicating that the rock is frequently vulnerable to dissolution (carbonate rock) creating secondary porosity.

In the AR/G (Fig. 20), the volume of shale diminishes towards the northwest, effective porosity increases in the same direction, and water saturation declines towards the northwest. The North-West direction may exhibit advantageous reservoir characteristics for both formations.

##### Depositional environment

The A/R G formation is characterized by the shallow marine environment (tidal flats and nearshores) of high wave and current energy that forms grainstone with coarser grain sizes and good sorting^17,53^. In Fig. 20, A/R G formation effective porosity increased toward the northwest which is the area of high energy settings. The core investigation conducted on wells Sira8-3 and Sitra8-15 indicated that well Sitra8-3 is characterized by a tidally-dominated environment, featuring predominant sand-dominated tidal flats and alternating genetic units of mud and sand-dominated tidal flats, and sitra8-15 is Nearshore marine environment, Shale and heterolithic (Mud, sand and shale)rocks make up the majority of sitra8–15, and this is line up with the contour map of A/R G.

### Correlation

**Fig. 21:**
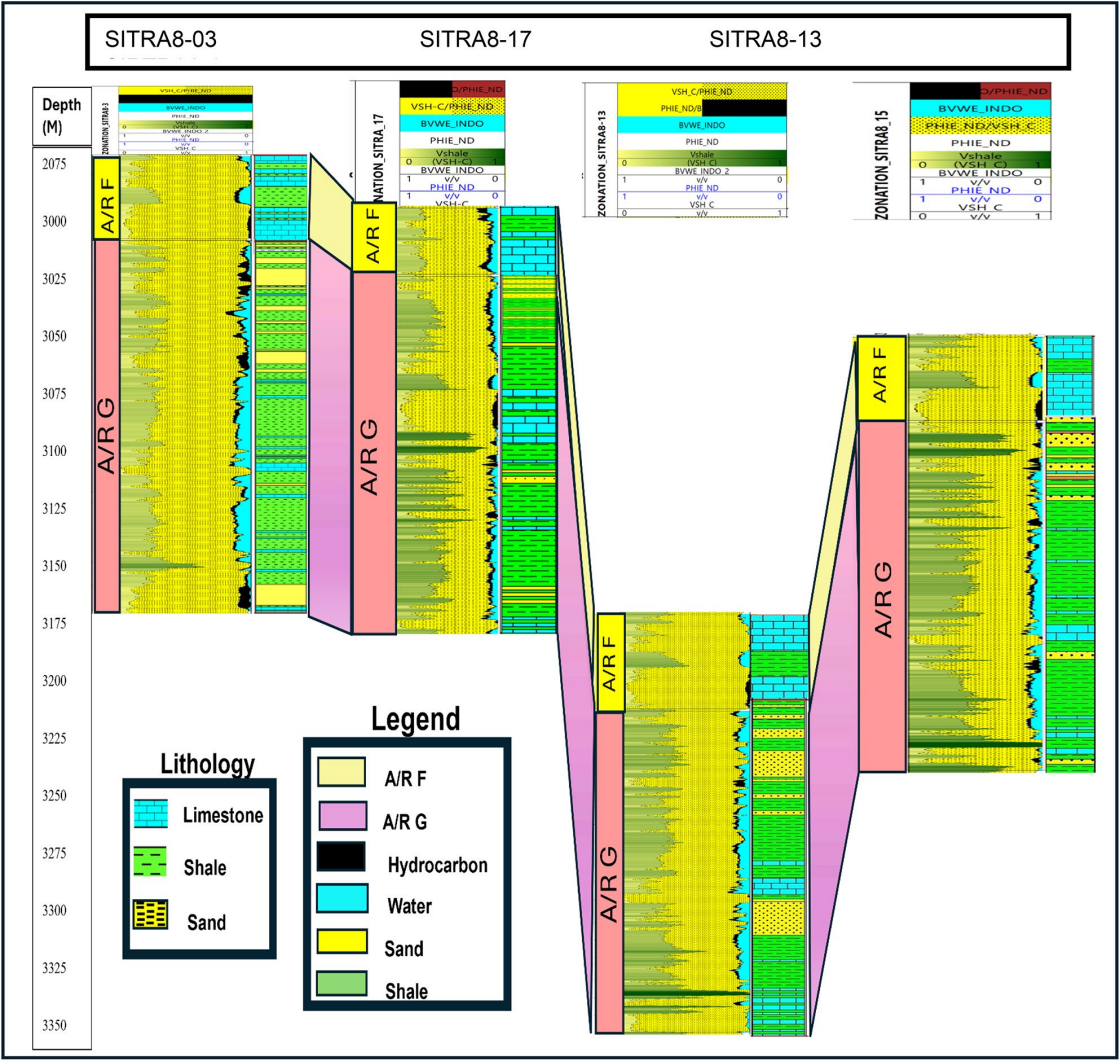
well log interpretation and correlation displaying the lithological and petrophysical variations of AR/F and AR/G in across the four wells.

Well Correlation (Fig. 21) entails the process of aligning well log signatures from different wells. Utilized for the purpose of selecting exploratory sites, assessing the distribution of net-to-gross ratio among wells, and detecting the stratification within a reservoir. It also provided evidence of the structural geometry of the reservoir^54^.

## Results and discussion

Table 3 displays the average values of the petrophysical parameters used to create contour maps (Figs. 19, 20) for shale volume, Porosity and water saturation of AR/F and AR/G.

The results demonstrate that well SITRA8-03 possesses ideal properties for a reservoir in both formations and drilling new development wells towards the northwest should be considered. The majority of the porosity in AR/F is attributed to the secondary porosity (Fig. 19b). Table 2 specifically in the form of fractures^11^. The value of the secondary porosity in SITRA8-03 is higher than the value in SITRA8-13 which makes SITRA8-03 the most promising well, the average porosity (6–14% of AR/G formation is illustrated in Fig. 20b. However, to provide greater clarity, the porosity levels in the upper and lower portions of AR/G vary between 8% and 10% and 10% and 17%, respectively. Sand interval is considered to be the critical factor, as indicated by the results. Badr EL-Din Company performed hydraulic fracturing operations in the Sitra8-3 and Sitra8-13 wells between 2008 and 2010, Targeting Lower AR/G to increase well productivity and, consequently, maximize rate and ultimate recovery; this led to the formation of fractured secondary porosity.

The correlation analysis that was performed between the litho-saturation models (rock models) for the four wells (Fig. 21), revealed that Well SITRA8-13 had an obvious structural depression compared to the other wells, indicating that it was drilled on a graben structure. AR/F formation is mainly composed of two carbonate intervals with a shale unit in-between and AR/G is composed of mainly shale and sand, and this has been verified in the outcome obtained from the cross plots (Figs. 6, 7), AR/F showed hydrocarbon saturation presence at the lower part in the four wells due to high organic content with (unconventional reservoir), While in AR/G only well SITRA8-03 has low volume of shale with a promising net pay hydrocarbon sand conventional reservoir interval at the upper part of AR/G and the hydrocarbon indicators results (Fig. 7) corroborate this.

In our research we missed the core data of AR/F formation However, the previous study indicates that the upper AR/F interval is more prone to brittleness compared to the lower section. While the lower AR/F interval has a higher concentration of organic matter. The petrographic investigation revealed the frequent occurrence of slender cracks and the existence of minor mechanical compaction. To commence production from this compact and unconventional carbonate reservoir, performing hydraulic fracking operations is strongly advised55 .The findings from the core analysis of the upper AR/G formation indicated that well sitra8-03 possesses a favorable porosity in the sand intervals, thereby validating the results obtained from all employed methodologies.

**Table 3:** Petrophysical analysis of AR/F and AR/G.

Well	Zones	Top	Bottom	Unit	Gross	Net	Av_Shale volume	Av_Effective porosity	Av_Water saturation	Av_Sec porosity
SITRA8-3	A/R F	2972	3007	M	35	32.35	0.136	0.046	0.519	0.089
SITRA8-3	AR/G	3008	3166	M	158	82.536	0.211	0.14	0.412	N/A
SITRA8-13	A/R F	3169	3210	M	41	35.9	0.192	0.048	0.466	0.021
SITRA8-13	AR/G	3211	3350	M	139	111.6	0.333	0.087	0.688	N/A
SITRA8-15	A/R F	3050	3084	M	34	25.912	0.218	0.046	0.52	N/A
SITRA8-15	AR/G	3085	3227	M	142	87.628	0.34	0.083	0.644	N/A
SITRA8-17	A/R F	2991	3020	M	29	28.26	0.249	0.112	0.62	N/A
SITRA8-17	AR/G	3021	3178	M	157	121.052	0.306	0.065	0.54	N/A

## Conclusion

This study intends to characterize the hydrocarbon potential, evaluate reservoir zones, and identify lithofacies through the exploitation of four wells drilled in sitra8 field Abu EL-Gharadiq basin based on our findings, the following conclusions can be stated:

1. From lithology cross plots, AR/G consists of sandstone (conventional reservoir) and shale while AR/F consists of limestone (unconventional reservoir) and shale.
2. Hydrocarbon indicators methods indicate that the upper and lower part of the AR/G formation in well sitra8-03 and the lower AR/F formation in the four wells display a hydrocarbon-bearing zone.
3. The computed Petrophysical variables in the Table 2 (shale volume, effective porosity and water saturation) showed that A/R is composed of limestone. The average secondary porosity in sitra8-03 is 8.9%. The same well in AR/G has the lowest shale, HC bearing zone, and good porosity, which agreed with the contour maps we created for (Vsh) porosity and water saturation. Figures 19 and 20 as the good reservoir quality increases towards the northwest direction where the sitra8-03 is located.
4. From the correlation, we can figure out that well sitra8-13 was drilled on a graben structural. This might potentially result in the entrapment of hydrocarbons at elevated well depths (sitra8-03 and sitra8-17).
5. Core analysis signifies a good porosity in the upper part of AR/G formation in well sitra8-03, and there is a thin fracture (secondary porosity) in AR/F formation in the same well.
6. All previous observations lead us to conclude that well SITRA8-03 is projected to yield the most and HC has the best reservoir attributes among the four wells.

## Data Availability

The data supporting the results of this study was obtained from the Egyptian General Petroleum Cooperation. However, there are limits on accessing these data since they were utilized under permission for this study and are not publicly available This data is available from the corresponding author upon reasonable request and with permission of (The Egyptian General Petroleum Cooperation).
